# A conserved opal termination codon optimizes a temperature-dependent trade-off between protein production and processing in alphaviruses

**DOI:** 10.1126/sciadv.ads7933

**Published:** 2025-04-18

**Authors:** Tamanash Bhattacharya, Eva M. Alleman, Tiia S. Freeman, Alexander C. Noyola, Michael Emerman, Harmit S. Malik

**Affiliations:** ^1^ Basic Sciences Division, Fred Hutchinson Cancer Center, Seattle, WA, USA.; ^2^ Department of Microbiology, University of Washington, Seattle, WA, USA.; ^3^ Human Biology Division, Fred Hutchinson Cancer Center, Seattle, WA, USA.; ^4^ Howard Hughes Medical Institute, Fred Hutchinson Cancer Center, Seattle, WA, USA.

## Abstract

Most mosquito-transmitted alphaviruses encode a premature opal termination codon upstream of their viral polymerase. We show that the Sindbis virus (SINV) opal codon outperforms other stop codons in primate cells at 37°C due to optimal translational readthrough. However, increased readthrough of all stop codons reduces opal preference at 28°C in primate and mosquito cells. Opal also outperforms all sense codons because opal-to-sense substitutions lead to excess polyprotein production at 37°C, disrupting orderly polyprotein processing and production of viral genomic RNAs (gRNAs) required for virus production. Increased readthrough at 28°C dampens the fitness advantages of opal codons. Unexpectedly, we find that a naturally occurring SINV mutation restores sense-codon fitness by further delaying polyprotein processing, allowing adequate time to produce gRNAs. Similar temperature-dependent mechanisms occur in the distantly related dual-host alphavirus, Ross River virus. Our work highlights sophisticated strategies dual-host alphaviruses use to optimize replication in divergent temperatures through a single codon.

## Teaser

Alphaviruses optimize viral polymerase production and polyprotein processing at distinct temperatures via a premature stop codon.

## INTRODUCTION

**Fig. 1.**
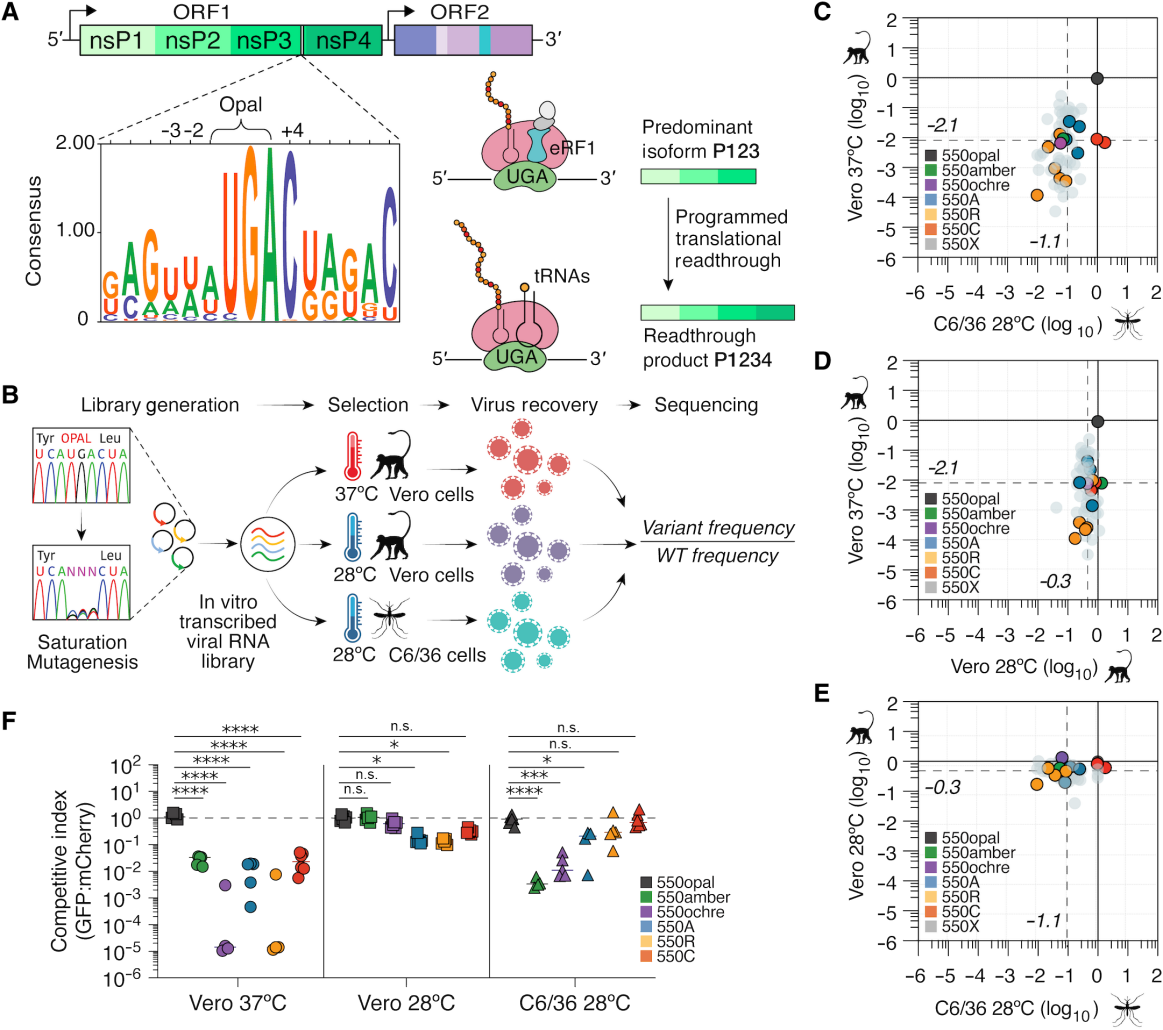
Saturation mutagenesis screens identify host- and temperature-specific constraints acting on SINV nsP3 opal codon. (**A**) Schematic of alphavirus nsP indicating the location of the opal stop codon in nsP3. Logo plot shows conservation at the opal stop codon site and its genomic context among dual-host alphaviruses. −3 and +4 positions indicate constrained residues that aid in the translational readthrough of the opal codon. Translational readthrough of the “leaky” opal stop codon leads to nsP4 (RdRp) expression and commencement of de novo viral RNA synthesis (left). ORF, open reading frame. (**B**) Schematic of saturation mutagenesis screen. The WT opal stop codon in SINV nsP3 was mutated to 1 of 64 possible codons using PCR mutagenesis. The mutant virus library was transcribed in vitro and subjected to selection by passaging through African green monkey (Vero) cells grown at 37° or 28°C and mosquito (C6/36) cells grown at 28°C. Following selection, viruses were sequenced alongside the input/preselection plasmid library to determine the relative selection of each codon variant relative to the WT opal codon. (**C** to **E**) Correlation plot of mean “selection scores” of codon variants across distinct environmental variables. Horizontal and vertical dashed lines depict the median selection scores for all non-opal codon variants. 550X refers to all sense codons excluding alanine, arginine and cysteine codons. (**F**) Competition assays between GFP-tagged variant and mCherry-tagged WT (550opal) SINV strains. Data presented are representative of two independent experiments with biological replicates (*n* = 3). Two-way ANOVA with Tukey’s multiple comparisons test. *P < 0.05; ***P < 0.001; ****P < 0.0001; n.s., not significant.

Alphaviruses are a broad genus of enveloped, single-stranded, positive-sense RNA viruses that occur nearly worldwide and profoundly affect human health (*1*, *2*). Most extant alphaviruses are obligate dual-host viruses, i.e., their transmission relies on obligate alternation between arthropod vectors and vertebrate hosts (*3*). Dual-host alphaviruses are subject to divergent selective pressures in insect and vertebrate hosts, resulting in viral genomic features that might be host specific (*4*, *5*). One such host-specific genome feature is the opal (UGA) termination codon that disrupts the nonstructural polyprotein (nsP) open reading frame in most alphaviruses (*6*). The nsP3 opal codon is located at the C-terminal end of the nonstructural protein 3 (*nsP3*) gene, just upstream of the nonstructural protein 4 (*nsP4*) (Fig. 1A) (*6*). First described in the Sindbis virus (SINV) genome, the opal codon is part of a type II programmed ribosomal readthrough (PRT) motif, which is characterized by the UGA codon followed by a cytosine (C), with a more modest preference for uracil (U) residues at the -2 and -3 positions (Fig. 1A). These flanking nucleotides promote efficient PRT, during which cellular tRNAs outcompete canonical eukaryotic translation termination factors such as eRF1 and incorporate a sense codon instead of the termination codon (Fig. 1A) (*7*, *8*). Through PRT, the *nsP* genes of most alphaviruses encode two distinct polyproteins (*9*). The first comprises nsP1-nsP2-nsP3 (P123). Ribosomal readthrough of the opal stop codon also produces lower amounts of the longer nsP1-nsP2-nsP3-nsP4 polyprotein (P1234) at a rate of ~5 to 20% (*10*). Because nsP4 is the viral RNA-dependent RNA polymerase (RdRp), sufficient PRT is required for de novo viral RNA synthesis.

Because nsP processing and nsP4 expression are often rate-limiting steps for alphavirus replication, the almost strict preservation of the nsP3 opal codon suggests that the opal codon must have been conserved because it confers a selective advantage. For example, previous analyses of SINV, Chikungunya virus (CHIKV), and O’nyong-nyong virus (ONNV) isolates with opal versus “sense” (nonstop) codons revealed notable differences in tissue tropism (CHIKV and SINV), virulence (CHIKV and SINV), and vector competence (ONNV) (*11*–*13*). Replacing the opal codon with sense codons significantly reduced CHIKV replication kinetics, host specificity, and virulence in mice (*12*). In vitro, passaging experiments with Eastern Equine Encephalitis Virus (EEEV), CHIKV, and ONNV further demonstrated the selective consequences of opal-to-sense codon mutations (*14*–*16*). For instance, experimental passaging of the alphavirus EEEV exclusively in mosquito cells resulted in the opal codon being replaced by sense codons in multiple independent lineages (*14*). In contrast, opal-to-sense codon substitutions were not observed following EEEV passaging in vertebrate cells (*14*). Similarly, Semliki Forest virus (SFV) and CHIKV variants encoding either sense codons (CGA, arginine) or alternate stop codons (UAG, amber; UAA, ochre) were less fit in vertebrate cells but more tolerated in mosquito cells (*17*, *18*). These and related studies led to the hypothesis that host-specific constraints have preserved the opal stop codon in alphaviruses. Yet, the exact nature of these constraints and how they affect alphavirus fitness remain unknown.

Here, we investigated the function and selective constraint on the nsP3 opal codon using comprehensive mutational scanning of this codon in the prototype alphavirus SINV in both primate (Vero) and mosquito (C6/36) cells. We find that the opal codon outperforms all other codon variants in Vero cells at 37°C, but this strong preference is reduced in both mosquito cells and Vero cells at 28°C (passaging temperature for mosquito cells). Thus, the primary determinant selecting for the opal stop codon is not host genetics but viral passaging temperature. We demonstrate that the opal codon is favored over the amber and ochre stop codons because it provides the highest translational readthrough and production of the nsP4 protein at 37°C. However, the replacement of the opal codon with sense codons produces too much full-length polyprotein (P1234), which impairs optimal nsP processing, leading to a delay in the switch between minus-strand and positive-strand production, which drastically reduces SINV replicative fitness at 37°C. A naturally occurring “suppressor” mutation in an SINV strain carrying a sense codon unexpectedly compensates for a delayed transition from minus to genomic RNA (gRNA) production by further delaying the subsequent transition between gRNA and subgenomic RNA (sgRNA) production. Using Ross River virus (RRV), a distantly related old-world alphavirus belonging to the SFV clade, we show that similar compensatory mechanisms required to maintain opal-to-sense mutations already exist across this clade.

Our studies reveal that the opal stop codon is the optimal solution to an inherent trade-off in alphavirus replication at 37°C. It produces enough nsP4 protein to maximize SINV replication without impairing nsP processing or the synchronized RNA replication transitions—minus-strand to genomic to sgRNA synthesis—required for optimal fitness. Our findings reveal the mechanistic basis of an exquisite strategy deployed by dual-host alphaviruses at a single codon to optimize fitness in disparate hosts at their native temperatures.

## RESULTS

### Host temperature imposes the predominant selection pressure for the nsP3 opal codon

To investigate the selective constraints acting on the in-frame opal codon site in alphavirus nsP3 (Fig. 1A) in divergent hosts and at different temperatures, we generated a saturation mutagenesis library of all possible mutations at the nsP3 opal stop codon site (codon 550) of the prototype alphavirus Sindbis (SINV). A similar strategy was previously used to identify host-specific constraints in other alphavirus proteins (*19*, *20*). Using in vitro transcription (IVT), we created replication-competent viral RNA from this library and transfected it either into Vero cells (derived from African green monkeys) maintained at 37°C (hereafter referred to as Vero-37°) or into C6/36 cells (derived from *Aedes albopictus* mosquitoes) maintained at 28°C (hereafter referred to as C6/36) (Fig. 1B). We deliberately chose vertebrate and mosquito cells with compromised innate immunity to remove the dominant effects of selective pressure imposed by vertebrate-specific type I/II interferon (IFN) or mosquito-specific RNA interference pathways (*21*, *22*). To further distinguish between the effects of temperature and host genetics, we also transfected the library into Vero cells at 28°C (hereafter referred to as Vero-28°) (Fig. 1B); reduced cell viability precluded us from testing the variant library in C6/36 cells at 37°C. Seventy-two hours posttransfection, we harvested viruses from cellular supernatants and conducted deep sequencing to determine “selection scores” for each SINV variant. These selection scores represent the ratios of variant frequency postselection to their initial frequencies in the input library, normalized to the wild-type (WT) opal codon (Fig. 1B; also see Materials and Methods). Thus, selection scores quantify the relative enrichment and depletion of SINV variants as indicators of their replicative fitness, with positive selection scores indicating enhanced fitness and negative selection scores indicating reduced fitness (Fig. 1B).

Consistent with its evolutionary conservation, we found that the WT opal codon variant significantly outperformed all other codon variants by more than 120-fold on average in Vero-37° (Fig. 1C and fig. S1, A and B). The mean selection score for all non-opal variants was −2.1 ± 1.1 (on a log_10_ scale). Most sense codons and alternate termination codons—amber (UAG) and ochre (UAA)—are significantly depleted. Selection scores for synonymous codons encoding the same amino acid were similar, indicating that selection primarily acts at the protein level rather than the underlying viral RNA sequence (fig. S1B). The opal codon remained the most preferred codon even in C6/36 cells, outperforming all codons by 12-fold on average (Fig. 1C); the cysteine sense codons are an exception and appear to be just as preferred as the opal codon. The mean selection score of −1.1 ± 0.47 (on a log_10_ scale) for all non-opal codons in C6/36 cells is much less severe than the mean selection score in Vero-37°. These results show that the selective constraint on the SINV opal codon is less stringent in mosquito cells than in vertebrate cells. We considered whether differential selection scores in Vero-37° versus C6/36 cells reflect differences in tRNA abundance between host cell types. However, selection scores do not appear to correlate with codon usage frequency in host cells (*Chlorocebus aethiops* versus *A. albopictus*; fig. S1C) (*23*, *24*).

The marked difference in selective constraint acting on opal stop in Vero-37° versus C6/36 cells could result from genetic or metabolic differences between the host cell types or passaging temperatures. A previous study showed that SFV variants with arginine sense codons had lower fitness in chicken cells at 39°C, but fitness could be restored at 30°C in the same cells (*18*). To test for temperature-dependent effects more comprehensively, we compared selective constraints acting on the opal codon in Vero-37° versus Vero-28° (Fig. 1D and fig. S1, A and B). In Vero-28° cells, we found that the mean selection score for all non-opal variants was only −0.34 ± 0.24 (on log_10_ scale). This represents the weakest preference for the opal codon among all three conditions tested. Selection for the opal codon is only twofold higher than non-opal variants. Several sense codons (including UGU for cysteine) and the amber stop codon (UAG) had nearly identical selection scores to the opal stop codon in Vero-28° (Fig. 1D and fig. S1, A and B). As a result, there are only modest differences in variant selection between C6/36 and Vero-28° cells (Fig. 1E). These findings strongly suggest that passaging temperature, rather than host cell type differences, is the predominant factor driving selective retention of the SINV nsP3 opal codon, although host cell differences also play an important role (Fig. 1E).

Next, we validated the selection scores obtained from our mutagenesis and pooled passaging strategy using two orthogonal assays. These assays measured either competitive (relative) or independent (absolute) fitness of six SINV variants encoding either of three alternate stop codons—opal, amber, or ochre—or three sense-codons—arginine (550R), cysteine (550C), or alanine (550A). In the first validation assay, we measured the fitness of green fluorescent protein (GFP)–tagged SINV variants relative to an mCherry-tagged WT SINV strain (550opal) using coinfections in Vero-37°, Vero-28°, or C6/36 cells. We harvested cells 48 hours postinfection (hpi) and analyzed them using flow cytometry to calculate the ratio of cells infected with the GFP-tagged variant versus mCherry-tagged WT SINV. We normalized the competitive index by comparing the ratio of GFP:mCherry cells in a control coinfection with GFP-tagged WT SINV (Fig. 1F). We then quantified the competitive fitness of the other five GFP-tagged SINV variants relative to the mCherry-tagged WT SINV. We found that the 550opal variant outcompetes 550amber and 550ochre by two to four orders of magnitude in Vero-37° and C6/36 cells, but the relative fitness of 550amber and 550ochre is almost fully restored in Vero-28°. Similarly, the high fitness advantage of the 550opal variant over 550R, 550C, or 550A in Vero-37° is considerably reduced in C6/36 cells or Vero-28°, with 550C identical in fitness to WT SINV in C6/36 cells.

To assess the independent fitness of the SINV variants, we compared their infection rates relative to WT SINV (550opal) in Vero-37°, Vero-28°, and C6/36 cells using flow cytometry (fig. S2). The results from this assay were also consistent with results from the pooled and pairwise competition assays (Fig. 1, C to F). The selection scores obtained from our pooled screening of all variants at *nsP3* codon 550 strongly correlate with individually measured competitive or absolute fitness measurements of the six selected SINV variants [Pearson *R* = 0.915, 95% confidence interval (CI) = 0.7824 to 0.9683, *P* value < 0.0001]. These results confirm that the pooled selection scores are reliable measures of viral fitness and that the WT SINV (550opal) significantly outperforms alternate stop and sense-codon variants in Vero-37°.

### Translational readthrough efficiency underlies fitness differences between alternate stop codons

We investigated why the opal stop codon is preferred over other stop codons—amber and ochre—in SINV nsP3. All SINV variants encoding stop codons require translational readthrough to produce the nsP4 RdRp necessary for viral replication. However, different stop codons have variable translational readthrough rates in eukaryotic mRNAs, with the opal codon exhibiting the highest readthrough frequency, followed by amber and then ochre (*25*, *26*). Because alphaviruses use host translation machinery, we reasoned that viral RNAs must be subject to similar translational preferences.

**Fig. 2.**
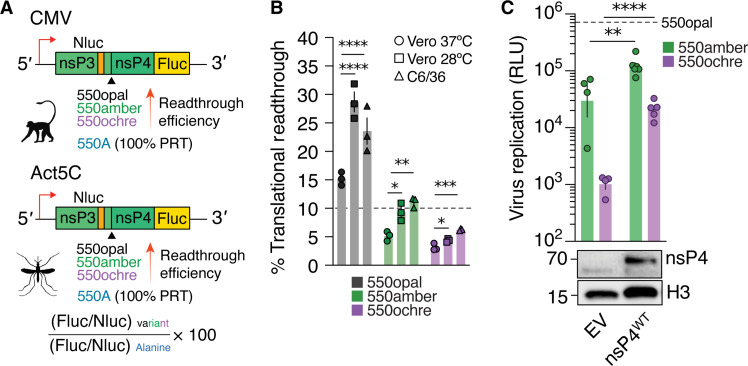
Temperature-dependent translation readthrough efficiency dictates the fitness of SINV variants encoding alternate stop codons. (**A**) SINV nsP3/4 translational readthrough was quantified using dual-luciferase reporter constructs carrying CMV promoters in vertebrate cells and Act5C promoters in insect cells. (**B**) Translational readthrough of variants containing alternate stop codons in Vero-37°, Vero-28°, or C6/36 cells. The horizontal dashed line denotes a 10% translational readthrough. Data presented are representative of two independent experiments with biological replicates (*n* = 3). (**C**) SINV variants carrying alternate stop codons were used to infect Vero-37° cells in the presence or absence [empty vector (EV)] of WT SINV nsP4 in trans. Each virus expresses an in-frame Nluc reporter, whose expression is directly proportional to virus replication. Replication of SINV carrying alternate stop codons was quantified 24 hpi in the presence and absence of exogenous nsP4 using Nluc expression as a readout. Dashed line represents WT (550opal) SINV replication. Data presented are representative of two independent experiments with biological replicates (*n* = 4 to 6). Two-way ANOVA with Tukey’s multiple comparisons test. *P < 0.05; **P < 0.01; ***P < 0.001; ****P < 0.0001. RLU, relative luminescence units.

We designed dual-luciferase reporter constructs to measure the amount of translational readthrough with different stop codons on nsP3 (Fig. 2A). The first reporter is Nano luciferase (Nluc) fused upstream of the codon 550 site to the *nsP3* hypervariable domain (HVD). The second reporter is Firefly luciferase (Fluc) fused to *nsP4*, downstream of the termination codon readthrough element (RTE) located 100 nucleotide downstream from the start of nsP4. This region has a structured RTE necessary for optimal readthrough (fig. S3, A and B) (*8*, *27*, *28*). We transfected these SINV P34 constructs, engineered to be driven by the cytomegalovirus (CMV) promoter-enhancer, in Vero-37° or Vero-28° cells and quantified Nluc and Fluc expression. We made analogous constructs driven by the Drosophila Actin (Act5C) promoter for experiments in C6/36 cells. We measured Fluc:Nluc ratios for all three stop codons in the SINV P34 construct 48 hours posttransfection to quantify translational readthrough efficiency, comparing Fluc:Nluc levels for a sense codon (alanine, GCA) that requires no translational readthrough. We found that the P34 construct encoding 550opal had ~15% translational readthrough (relative to the sense codon) in Vero-37° (Fig. 2B); these levels accord well with previous measurements of 5 to 20% (*10*). In contrast, translational readthrough for the P34-550amber (5%) and P34-550ochre constructs (2%) was significantly lower in Vero-37°. Western blot analyses of infected Vero-37° cells confirmed lower nsP4 expression in the amber and ochre variants (fig. S3C), consistent with the Fluc:Nluc reporter assay. These results demonstrate a hierarchy in translational readthrough efficiency of the three termination codons within the context of the alphavirus genomic RNA.

We found that translational readthrough efficiency for all stop codon variants was higher in Vero-28° and C6/36 cells. However, the hierarchy in translational readthrough efficiency (opal > amber > ochre) remained true under all conditions (Fig. 2B and fig. S3B). These results confirm that the disparities in replicative fitness observed between the WT (550opal) and the alternate stop codon variants 550amber and 550ochre) can be attributed to lower translational readthrough frequencies, which lead to reduced nsP4 protein expression. This issue is particularly pronounced at 37°C but is improved at 28°C, where higher readthrough rates for the amber and ochre codons partially rescue fitness defects. Furthermore, given that infection rates of 550amber are nearly at WT levels in Vero-28° cells (fig. S2), we conclude that a threshold level of ~10% readthrough efficiency is sufficient to restore SINV fitness in vertebrate cells (Fig. 2B). However, a higher readthrough frequency might be required to achieve WT levels of SINV fitness in mosquito cells, given the inherent instability of nsP4 (*29*). Moreover, the translational readthrough of the amber and ochre codons (Fig. 2B) is not entirely predictive of their relative fitness in C6/36 cells (Fig. 1F and fig. S2), suggesting that additional readthrough-independent host-specific factors in mosquito cells might contribute to viral fitness.

If replication defects of alternate stop codon SINV variants in Vero-37° were primarily due to insufficient nsP4 expression, we hypothesized that additional, exogenous nsP4 production might improve replicative fitness of 550amber and 550ochre SINV variants. We tested this hypothesis by cotransfecting a WT nsP4-expressing construct with the SINV variants. Consistent with our expectation, we found that exogenous nsP4 expression significantly enhanced the replication of 550amber and 550ochre SINV variants (Fig. 2C). Our findings demonstrate that lower translational readthrough and nsP4 production are the predominant cause of lower fitness of alternate stop codons at 37°C, which is naturally ameliorated by higher translational readthrough at 28°C. This trend of temperature-dependent tolerance of alternate stop-codon variants (550amber and 550ochre) was also observed across multiple vertebrate cell lines (fig. S4).

### Fitness loss of sense-codon SINV variants is due to delayed plus-strand (genomic) RNA synthesis

Our findings that reduced translational readthrough and expression of the alphavirus nsp4 lowers SINV fitness for non-opal stop codons appear to be at odds with our observation that sense-codon SINV variants exhibit lower fitness. Sense-codon variants should produce higher levels of nsP4 than opal because they do not depend on translational readthrough (Fig. 1, D and F, and fig. S3). However, a further increase in nsP4 expression in the event of an opal-to-sense mutation is likely to be offset by the short half-life of nsP4, given its rapid turnover via the ubiquitin-dependent proteolysis pathway (*29*–*31*). Thus, the mechanistic basis of fitness loss in sense-codon SINV variants in Vero-37°, which must differ fundamentally from that for the ochre and amber SINV variants.

**Fig. 3.**
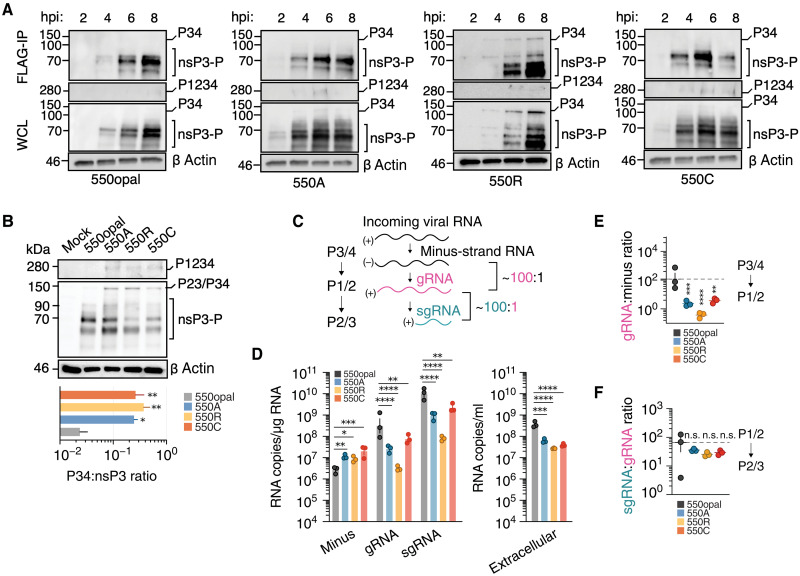
Aberrant polyprotein processing reduces the fitness of sense-codon SINV variants. (**A**) Vero-37° cells were infected (MOI = 5) with WT (550opal) or sense-codon (550A, 550R, or 550C) SINV variants expressing 3XFLAG-tagged nsP3. At indicated times postinfection, total protein (whole-cell lysate (WCL)] was subjected to IP using anti-FLAG (FLAG-IP) to enrich for unprocessed and processed nsP3. Blots were probed with anti-FLAG polyclonal and anti-β actin monoclonal antibodies. (**B**) Vero-37° cells were infected (MOI = 5) with WT (550opal) or sense-codon (550A, 550R, or 550C) SINV variants expressing 3XFLAG-tagged nsP3. At 18 hpi, total protein was extracted for Western blotting. Blots were probed with anti-FLAG polyclonal and anti-β actin monoclonal antibodies. Data are representative of three independent experiments. (Bottom) Ratio of unprocessed P34 to processed nsP3 in infected cells. (**C**) The current model of alphavirus nsP processing summarizes the sequential processing steps catalyzed by the alphavirus nsP2 protease, which are required to form distinct processing intermediates that synthesize specific viral RNA species. Under optimal replication conditions, gRNA:minus-strand and sgRNA:gRNA ratios are expected to be 100:1. (**D**) Levels of SINV RNA species in Vero-37° cells (*n* = 3). Cells were infected (MOI = 0.1) and harvested to quantify levels of minus-strand (4 hpi), genomic (gRNA, 18 hpi), subgenomic RNA (sgRNA, 18 hpi), and extracellular genomic RNA (18 hpi). (**E**) Ratio of SINV genomic (gRNA) to minus-RNA levels in Vero-37°. (**F**) Ratio of subgenomic (sgRNA) to genomic (gRNA) RNA species. Two-way ANOVA with Tukey’s multiple comparisons test. *P < 0.05; **P < 0.01; ***P < 0.001; ****P < 0.0001; n.s., not significant.

Both P123 and P1234 polyproteins undergo proteolytic processing by the nsP2 protease (nsP2^Pro^) through a series of highly orchestrated steps (Fig. 3A) as the nsP2^Pro^ sequentially transitions its cleavage site preference from P3/4 to P1/2 to P2/3 (*10*). Because sense-codon variants produce 85% more P3/4 substrate than WT SINV (Fig. 2B), we hypothesized that excess P1234 production might delay this orderly transition, with deleterious consequences on viral fitness.

We conducted Western blot analyses of Vero-37° cell lysates to test this hypothesis. We used a FLAG epitope-tagged nsP3 SINV strain, which allowed us to visualize nsP3 and any nsP3-containing processing intermediates using an anti-FLAG antibody. Processed nsP3 could be readily visualized under all conditions. Processed nsP3 appeared 4 hpi in WT SINV cells and increased over time. In contrast, processed nsP3 in 550A- and 500C-variant-infected cells could be detected by 2 hpi, likely reflecting their faster replication kinetics during early infection. By 4 hpi, higher levels of unprocessed P1234 could be detected in sense-codon variant-infected cells compared to cells with WT SINV (Fig. 3A and fig. S5C). This is partly expected due to higher P1234 production in sense-codon variants. However, it may also reflect delayed polyprotein processing. In support of this possibility, we also detected the appearance of an aberrant processing product, P34, as early as 4 hpi in sense-codon variants, most prominently in cells infected with 550R. In contrast, the aberrantly processed P34 product was absent in cells with WT SINV at all time points tested between 2 and 8 hpi. We confirmed the identity of this processing product by immunoprecipitation (IP) of FLAG-tagged nsP3 (Fig. 3A, top lanes) whenever possible. These results suggest that sense-codon SINV variants experience polyprotein processing defects that are sometimes triggered at the onset of infection and persist over time. For 550C, this is also supported by the observation that processed nsP3 levels reduce between 6 and 8 hpi (Fig. 3A and fig. S5C).

To test the persistence of nsP processing defects, we conducted Western blot analyses of sense-codon SINV variants in Vero-37° cell lysates 18 hpi to identify aberrations or delays in P1234 processing in sense-codon SINV variants (Fig. 3B). In addition to anti-FLAG, we used two polyclonal antibodies previously raised against SINV nsP2 and nsP4 proteins (fig. S6A); no suitable anti-nsP1 antibody was available. Western blot analyses with all three reagents showed no discernible differences in the steady-state levels of nsP4 for any of the SINV strains passaged in Vero-37° (fig. S6A). In contrast, levels of nsP3 (which is heavily phosphorylated) were quantifiably lower in some sense-codon SINV variants in Vero-37° cells (fig. S6G). A similar trend was also seen with processed nsP2. Our findings reveal that the lower fitness of sense-codon variants is not caused by changes in polymerase (nsP4) levels but by lower levels of processed nsP2 and nsP3; our findings are consistent with previous reports (*12*, *30*).

Our Western blot analyses revealed significant evidence of delayed or aberrant polyprotein processing in Vero-37° cell lysates from sense-codon SINV infections relative to WT SINV (Fig. 3C). Using anti-FLAG and anti-nsP2 antibodies, we observed higher levels of the unprocessed P1234 polyprotein (280 kDa) in lysates from sense-codon SINV variants (Fig. 3, A and B, and fig. S5C). In addition, we observed aberrant P12 and P34 products in the nsP2, nsP3, and nsP4 blots of sense-codon SINV variants. Higher accumulation of unprocessed P34 could also be readily observed independently in the nsP3-FLAG and nsP4 blots (Fig. 3, A and B, and fig. S6, A, D, and H). Similarly, the presence of P12 in Vero-37° lysates suggests that P1/2 cleavage is also delayed or disrupted in sense-codon SINV variants (fig. S6A). P12 and P34 intermediates can only be generated if the P2/3 cleavage aberrantly precedes the P3/4 and P1/2 cleavage steps (*32*). The high levels of P34 and P1234 in sense-codon SINV variants in Vero-37° contrast with nearly undetectable levels of these aberrant products in WT SINV (Fig. 3, A and B, and figs. S5C and S6, A, D, and H). Our findings reveal that P3/4 cleavage is delayed despite the presence of proteases capable of cleaving this site (*32*). Collectively, our Western blot analyses reveal nsP processing defects among sense-codon SINV variants in Vero-37°.

Sequential and timely cleavage of the nsP (P1234) is critical for synthesizing different viral RNA species in proportional amounts required for viral replication (Fig. 3C) (*10*, *32*, *33*). Initial P1234 cleavage at the P3/4 site, located six amino acids downstream of the opal codon, gives rise to P123 and nsP4, constituting the replicase synthesizing the minus-strand RNA (*34*). The second cleavage reaction at the P1/2 site releases nsP1, P23, and nsP4, which irreversibly stops viral minus-strand RNA synthesis (*10*, *33*, *35*). The third and final cleavage reaction occurs at the P2/3 site, giving rise to fully processed nsP1, nsP2, nsP3, and nsP4 proteins, which together are responsible for producing predominantly sgRNA from a downstream promoter element on the minus-strand RNA (*36*). Thus, cleavage of the nsP is closely tied to changes in RNA synthesis from minus-strand to gRNA and from gRNA to sgRNA (Fig. 3A). We next examined viral RNA levels using reverse transcription quantitative polymerase chain reaction (RT-qPCR) in Vero-37° cells infected with either WT SINV or each of three sense-codon SINV variants (550A, 550R, or 550C) to infer how differences in polyprotein processing affected the synthesis of different intracellular viral RNA species (Fig. 3D). We were motivated by the observation that processed nsP3 levels were significantly reduced for some sense-codon variants later during infection, concurrent with an increase in unprocessed P1234 and P34 (Fig. 3B and figs. S4 and S6H). At 4 hpi, we found that minus-strand levels were two to threefold higher than WT SINV for all three sense-codon SINV variants in Vero-37° (Fig. 3D), suggesting elevated abundance or activity of the unprocessed minus-strand replicase P123. At 18 hpi, we found that gRNA levels were significantly lower for sense-codon SINV variants than WT SINV (Fig. 3D). As a result, whereas WT SINV (550opal) maintains the typical ratio of ~100 gRNA copies per minus strand (*37*, *38*), sense-codon SINV variants have significantly lower gRNA, only making ~1 gRNA copy per minus strand (Fig. 3E). We also measured sgRNA levels in WT and sense-codon SINV variants to evaluate whether the processing defects persist or worsen in downstream steps. At 18 hpi, we found that sgRNA levels were also significantly lower in sense-codon SINV variants than in WT SINV (Fig. 3D). However, all four SINV strains had comparable sgRNA:gRNA ratios of ~50 to 100, typical of WT SINV (Fig. 3F) (*37*, *38*).

Our findings suggest that the transition from minus-strand to gRNA replication, which occurs upon P1/2 cleavage, is significantly delayed by the overproduction of the P1234 polyprotein in sense-codon SINV variants, resulting in higher minus-strand and much lower gRNA production. This reduction in gRNA production directly translates to fewer extracellular genome copies of SINV, i.e., lower total and infectious virus production 24 hpi in sense-codon SINV variants than WT SINV (Fig. 3D and fig. S7A). We also infer that P2/3 cleavage is not delayed in the sense-codon SINV variants because sgRNA:gRNA ratios are typical (Fig. 3F). Thus, our Western blot analyses and RNA species profiling implicate aberrant polyprotein processing and a delayed switch from minus-strand to genomic RNA replication as the primary causes of the reduced fitness of sense-codon SINV variants in Vero-37°.

### Lower temperatures reduce the fitness advantage of opal codon over sense-codon SINV variants

**Fig. 4.**
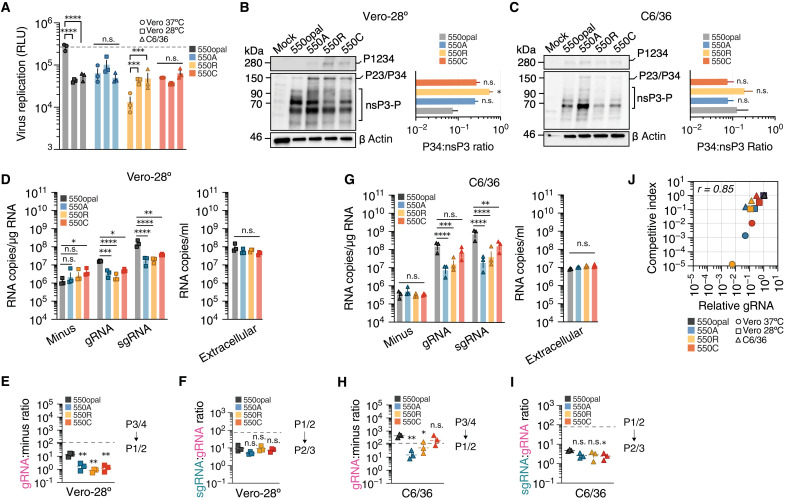
Relative fitness gap between WT and sense-codon SINV variants are reduced in mosquito cells. (**A**) Replication of WT (550opal) and sense-codon (550A, 550R, or 550C) SINV variants expressing translationally fused luciferase reporter in Vero-37°, Vero-28°, and C6/36 cells (*n* = 3). Cells were transfected with 100 ng of capped, in vitro–transcribed viral RNA, and viral replication was measured 24 hours later using luciferase activity. (**B** and **C**) Vero-28° (B) and C6/36 (C) cells were infected (MOI = 5) with WT (550opal) or sense-codon (550A, 550R, or 550C) SINV variants expressing 3XFLAG- tagged nsP3. At 18 hpi, total protein was extracted for Western blotting. Blots were probed with anti-FLAG polyclonal and anti-β actin monoclonal antibodies. Data are representative of three independent experiments. (Right) Ratio of unprocessed P34 to processed nsP3 in infected cells. (**D**) Levels of SINV RNA species in Vero-28° cells (*n* = 3). Cells were infected (MOI = 0.1) and harvested to quantify levels of minus-strand (4 hpi), genomic (gRNA, 18 hpi), subgenomic RNA (sgRNA, 18 hpi), and extracellular genomic RNA (18 hpi). (**E** and **F**) Ratio of genomic (gRNA) to minus viral RNA species (E) and subgenomic (sgRNA) to genomic (gRNA) RNA species (F) in Vero-28° cells. (**G**) Levels of SINV RNA species in C6/36 cells (*n* = 3). Cells were infected (MOI = 0.1) and harvested to quantify levels of minus-strand (4 hpi), genomic (gRNA, 18 hpi), subgenomic RNA (sgRNA, 18 hpi), and extracellular genomic RNA (18 hpi). (**H** and **I**) Ratio of genomic (gRNA) to minus viral RNA species (H) and subgenomic (sgRNA) to genomic (gRNA) RNA species (I) in C6/36 cells. (**J**) Pearson’s correlation between variant competitive index (Fig. 1F) and relative gRNA levels. *r*^2^ = 0.73, 95% CI = 0.5469 to 0.9580, *P* = 0.0004. Two-way ANOVA with Tukey’s multiple comparisons test. *P < 0.05; **P < 0.01; ***P < 0.001; ****P < 0.0001; n.s., not significant.

The competitive fitness of sense-codon SINV variants is recovered at 28°C (Fig. 1F and fig. S2). A partial explanation for this recovery may stem from previous studies, which found that SINV replication kinetics are slower in mosquito cells at 28°C than in vertebrate cells at 37°C (*39*, *40*). To test whether slower replication of WT SINV (550opal) at 28°C could explain the relative fitness gain of sense-codon SINV variants, we first measured the replicative fitness of individual SINV variants across Vero-37°, Vero-28°, and C6/36 cells. At 24 hpi, we found that replication of WT SINV (550opal) in Vero-28° and C6/36 cells was approximately fivefold lower compared to Vero-37° (Fig. 4A). In contrast, the replication of sense-codon variants was comparable across all three conditions. These results strongly indicate that sense-codon variants are better competitors against WT SINV at 28°C because WT SINV replicates slower at this temperature. We observed a consistent trend of temperature-dependent increase in sense-codon variant infection rates (550A, 550R, and 550C) across several different vertebrate cell types (figs. S2 and S4). Although temperature-dependent recovery was most evident in nonhuman primate–derived cells (Vero and BSC40), this trend was relatively modest in human-derived cells [Huh7 and human embryonic kidney (HEK) 293T cells], suggesting that additional host-intrinsic factors might influence the fitness of different sense-codon SINV variants (Fig. 4A and figs. S2 and S4).

We next investigated the cadence of nsP synthesis and processing in Vero-28° cells. IP followed by Western blot analyses at early time points revealed subtle differences in nsP3 expression and processing between Vero-37° and Vero-28° cells. The most notable difference is that processed nsP3 levels were comparable across WT and sense-codon variants in Vero-28° (Fig. 4B and fig. S5D), unlike Vero-37° cells (Fig. 3B and fig. S5C). Aberrant processing products P1234 and P34 were still higher in the sense-codon variants versus WT SINV-infected cells between 6 and 10 hpi (fig. S5, A and D). However, Western blot analysis from samples collected later (18 hpi) during infection revealed aberrantly processed P34 in all samples, with only marginally higher P34:nsP3 ratios in sense-codon SINV variants than WT SINV (550opal) (Fig. 4B and fig. S6, B, E, and H). These observations can be partly attributed to higher levels of translational readthrough of the opal codon in WT SINV at lower temperatures (see Fig. 2B), which would result in increased P1234 production and concomitant processing delays akin to sense-codon variants. Furthermore, compared to Vero-37°, P12 was undetectable for WT (550opal) and all sense-codon SINV variants in Vero-28°, indicating at least a partial rescue of P1/2 processing defects (fig. S6, A and B).

Consistent with these Western blotting results, we found that minus-strand RNA levels for 550A or 550R did not significantly differ from WT SINV in Vero-28° (Fig. 4D). However, minus-strand levels were still marginally higher in 550C than WT SINV. Moreover, gRNA and sgRNA levels were generally lower for all SINV variants, including WT SINV, in Vero-28°. Thus, both WT and sense-codon SINV variants exhibit low gRNA:minus-strand RNA and sgRNA:gRNA ratios in Vero-28° (Fig. 4, E and F). For example, WT SINV produces 10-fold fewer gRNA molecules per minus-strand RNA in Vero-28° (Fig. 4E) than in Vero-37° (Fig. 3E). Consequently, gRNA:minus-strand RNA ratios of sense-codon SINV variants are relatively less impaired (~10-fold lower) than WT SINV in Vero-28° (Fig. 4E), whereas they are 100-fold lower in Vero-37° (Fig. 3E). Together, our experiments suggest that, unlike in Vero-37°, the replication of WT SINV is lowered to a degree such that it produces gRNA and extracellular virions at levels comparable to sense-codon variants in Vero-28° (Fig. 4, D and J). This trend is also recapitulated upon quantification of infectious viruses; we find that titers of SINV sense-codon variants are only 5- to 10-fold lower than WT SINV in Vero-28°, compared to 300- to 100,000-fold lower in Vero-37° (fig. S7).

Next, we turned our attention to C6/36 mosquito cells. IP–Western blot analysis showed the presence of processed nsP3 starting at 4 to 6 hpi in 550opal, 550A, and 550R-infected cells and at 8 hpi in 550C-infected cells (fig. S5, B and E). Unlike Vero cells, unprocessed P123 was observed in WT and sense-codon variant cells at all early time points between 4 and 10 hpi, indicative of a slower polyprotein processing rate in mosquito cells. However, we did not observe accumulation of unprocessed P1234 or P34 in any of the infected cells by 10 hpi, except for a high–molecular weight band corresponding to P23 (≥150 kDa) in 550C-infected cells (fig. S5, B and E). By 18 hpi, we could observe either no (550C) or only subtle (550A and 550R) differences in nsP synthesis and processing between WT (550opal) and sense-codon SINV variants (Fig. 4C). Most nsP3 appeared present in processed form, except for some P34 that was occasionally detected in cells infected with sense-codon SINV variants (Fig. 4C and fig. S6, C and F to H).

In C6/36 cells, profiling of viral RNA levels revealed little to no differences between sense codon variants and WT SINV in minus-strand RNA levels (Fig. 4G) or sgRNA:gRNA ratios (Fig. 4I). Although gRNA levels and gRNA:minus-strand RNA ratios were equivalent between the 550C variant and WT SINV, both 550A and 550R showed significantly lower gRNA levels and gRNA:minus-strand RNA ratios than WT SINV (Fig. 4, G and H). However, unlike in Vero-37° or Vero-28°, even the impaired 550A and 550R SINV variants produced higher amounts of gRNA than minus-strand RNA (≥10:1) in C6/36 cells (Fig. 4H). Because they serve multiple roles during infection, from minus-strand RNA synthesis to nsP expression to virion production, gRNA levels are rate limiting for viral fitness (Fig. 1F and fig. S2). This enhancement of intracellular gRNA in C6/36 cells completely restores WT levels of extracellular viral RNA in sense-codon variants despite reduced sgRNA levels (Fig. 4G). Corresponding levels of infectious virus production are also unaffected in most sense-codon SINV variants in C6/36 cells, except for 550R (fig. S7). These results explain why cysteine (550C) is the most tolerated amino acid substitution in C6/36 cells because it can produce WT levels of gRNA (Fig. 1, C, E, and F, and figs. S2 and S7). However, our findings do not fully explain why there are still marked differences between the different sense-codon variants (550C versus 550R for example).

Our findings suggest that the higher relative fitness of sense-codon variants in Vero-28° and C6/36 cells can be explained mainly by the slower replication kinetics of WT SINV (550opal). As a result, WT and sense-codon variants produce equivalent quantities of extracellular viral RNA, thereby reducing the fitness gap between WT and sense-codon SINV variants at 28°C (Figs. 1, E and F, and 4, D, G, and J).

### A naturally co-occurring mutation can rescue 550C SINV variant fitness

**Fig. 5.**
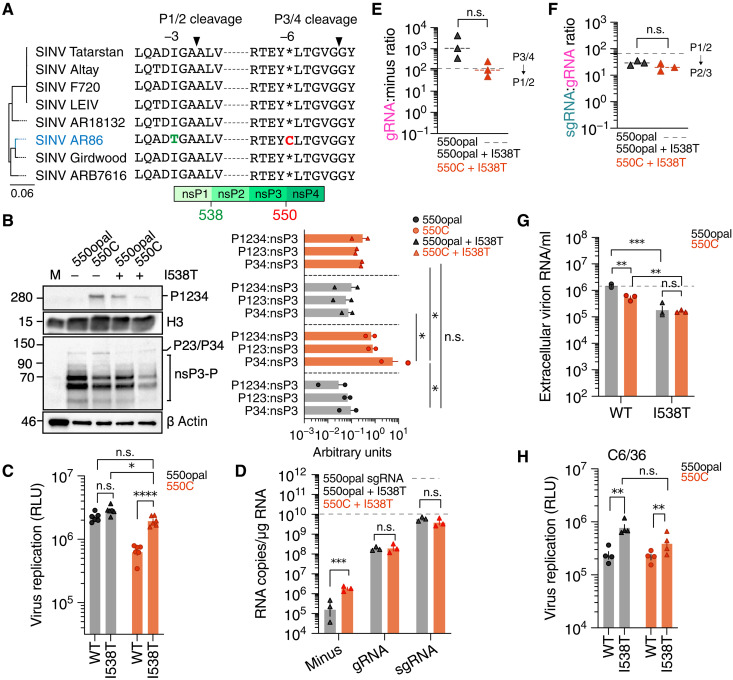
An nsP1 mutation can rescue the fitness defect of the 550C SINV variant in Vero-37°. (**A**) Maximum likelihood tree and multiple sequence alignment of the P1/2 and P3/4 cleavage sites in representative SINV strains (SINV AR86 is in blue). (**B**) WT (550opal) or 550C SINV variants expressing 3XFLAG- tagged nsP3, with or without the nsP1 I538T mutation, were used to infect Vero-37° cells (MOI = 5). At 18 hpi, total protein was extracted for Western blotting and probed with anti-FLAG, anti-H3 and anti-β actin antibodies. Data are representative of two independent experiments. (Right) Ratio of unprocessed (P1234, P123, and P34) to processed nsP3. (**C**) I538T variant replication in Vero-37° cells (*n* = 3), transfected with in vitro–transcribed RNA of WT or I538T mutation-carrying, 550opal or 550C SINV Nluc variants. Virus replication was assessed using luciferase assay 48 hours posttransfection. (**D**) Infected Vero-37° (MOI = 0.1) cells were harvested to quantify levels of minus-strand RNA (4 hpi), genomic RNA (gRNA, 18 hpi), and subgenomic RNA (sgRNA, 18 hpi) (*n* = 3). The horizontal dashed line denotes sgRNA copies produced by WT SINV (550opal). (**E** and **F**) Ratio of genomic to minus-strand (E) and subgenomic to genomic (sgRNA:gRNA) (F) viral RNA species in Vero-37° (*n* = 3). The dashed lines represent ratios calculated for WT SINV (550opal). (**G**) Virus production from Vero-37° cells infected with WT (550opal) or 550C SINV variants, with or without I538T mutation (MOI = 0.1). Extracellular viral genome copies were quantified using RT-qPCR from supernatants collected 18 hpi. (**H**) I538T variant replication in C6/36 cells (*n* = 3), transfected with in vitro–transcribed RNA of WT or I538T mutation-carrying, 550opal or 550C SINV Nluc variants. Virus replication was assessed using luciferase assay 24 hours posttransfection. Two-way ANOVA with Tukey’s multiple comparisons test. *P < 0.05; **P < 0.01; ***P < 0.001; ****P < 0.0001; n.s., not significant.

In contrast to every other SINV isolate found in nature, the neurovirulent SINV-like virus AR86 naturally encodes a cysteine codon (UGC) instead of the opal stop codon (*41*). We hypothesized that one or more co-occurring mutations in SINV-AR86 might rescue the fitness of the 550C SINV variant in Vero-37° by restoring the proper processing of the nsP P1234. Among several nonsynonymous mutations in SINV-AR86, we were especially intrigued by the I538T mutation near the P1/2 cleavage site motif in nsP1 (Fig. 5A). This I538T mutation has previously been shown to antagonize the JAK/STAT (Janus kinase–signal transducer and activator of transcription) pathway, preventing the induction of type I/II IFN responses, explaining why it may have been selected in vertebrate hosts (*42*, *43*). However, a previous study also showed that the I538T mutation caused delayed cleavage at the P1/2 site (*44*). This finding appeared to contradict our model because slower P1/2 cleavage would be predicted to exacerbate rather than ameliorate P1234 processing defects. However, slower P1/2 cleavage could also influence the efficiency of the downstream P2/3 cleavage. Previous in vitro studies have demonstrated that nsP1-containing proteases, such as uncleaved P123 and P12, cannot cleave at the P2/3 site (*32*). Therefore, we hypothesized that introducing the I538T mutation into SINV 550C might restore proper nsP processing by decelerating both the P1/2 and P2/3 cleavage steps.

Western blot analyses of infected cell lysates collected at 18 hpi supported this “dual delay” hypothesis, revealing higher than WT ratios of unprocessed P1234 to processed nsP3 (P1234:nsP3) in 550C+I538T (Fig. 5B and fig. S8). As expected, the P1234:nsP3 ratio was also elevated in cell lysates infected with 550C (Figs. 3B and 5B and fig. S5C). Unexpectedly, the P1234:nsP3 ratio also appeared slightly higher in 550opal+I538T-infected cells than WT SINV-infected cells (Fig. 5B). Consistent with our hypothesis, reduced P2/3 processing in the presence of the I538T mutation also significantly reduced levels of aberrantly processed P34 product in 550C, lowering the P34:nsP3 ratio down to WT (550opal) level (Fig. 5B and fig. S8). However, consistent with prior observations in SINV AR86, consecutive delays in P1/2 and P2/3 processing resulted in reduced levels of processed nsPs in 550C+I538T (fig. S8) (*44*).

Having confirmed P1/2 and P2/3 processing, we next quantified the replication of WT SINV (550opal) and 550C in the presence and absence of the I538T mutation in Vero-37° cells. In contrast to 550C, replication of 550C+I538T was significantly higher and nearly restored to the level of WT SINV (550opal) (Fig. 5C). However, in line with lower levels of processed nsP3, 550C+I538T replication was marginally lower than 550opal+I538T (Fig. 5C and fig. S8). Viral RNA profiling in Vero-37° cells revealed that 550C+I538T accumulated more minus-strand RNA than 550opal+I538T, presumably due to higher levels of unprocessed minus-strand replicase at early stages of infection (Fig. 5, B and D). However, both I538T variants produced equivalent levels of plus-strand gRNA and sgRNA (Fig. 5D). Consistent with our expectation that increased gRNA synthesis results in higher replicative fitness, absolute gRNA levels and the ratio of gRNA to minus-strand RNA in 550C+I538T-infected cells were comparable to WT SINV (550opal) levels (Figs. 3E and 5E). At the same time, we found that 550opal+I538T and 550C+I538T mutants produced less sgRNA than WT SINV—also expected given that the I538T mutation delays the switch from gRNA to sgRNA production (Fig. 5D). This is reflected in lower sgRNA to gRNA ratios for 550opal+I538T and 550C+I538T compared to WT SINV (550opal) (Figs. 3G and 5F). Thus, 550C SINV variants compensate for a delayed transition in gRNA production by further delaying the transition from gRNA to sgRNA production, enabling sufficient time for gRNA synthesis.

Reduced sgRNA levels in I538T mutants are not inconsequential; they lead to lower virus production, likely due to insufficient structural protein expression from a reduced sgRNA pool. Thus, although 550opal+I538T and 550C+I538T SINV variants produced equivalent amounts of extracellular genomic RNA or progeny virions 18 hpi, these levels were still lower in I538T mutants than either WT SINV or 550C (Fig. 5G). Intriguingly, 550opal+I538T and 550C+I538T replicate marginally better than their non-I538T counterparts in C6/36 cells (Fig. 5H), suggesting that sense-codon SINV variants might have selective advantages that allow them to thrive in mosquito vectors but are disadvantaged in vertebrate cells unless they acquire compensating mutations like I538T.

### Mechanism of sense codon tolerance is conserved across alphaviruses

In contrast to SINV, other old-world alphaviruses, particularly those within the SFV clade, exhibit high rates of opal-to-sense mutations (fig. S9). We investigated whether we could apply the insights gleaned from our SINV analyses to explain this occurrence. Given the requirement of P1/2 cleavage site residues toward tolerating opal-to-sense mutations in SINV, we next wondered whether similar mechanisms might explain sense-codon variants in the SFV clade. The P1/2 cleavage motif is highly conserved across members of the SFV clade (fig. S10). Previous findings suggest that weakening the P1/2 cleavage site in WT SFV, which encodes an arginine codon at the opal site, markedly reduces virus infectivity of both opal and sense-codon SFV variants in vertebrate cells (*18*). These observations led us to hypothesize that the conserved P1/2 cleavage motif among SFV clade viruses might help tolerate opal-to-sense mutations like the I538T mutation in SINV. This might explain the unusually high proportion of sense codon mutations in the SFV clade (fig. S10).

**Fig. 6.**
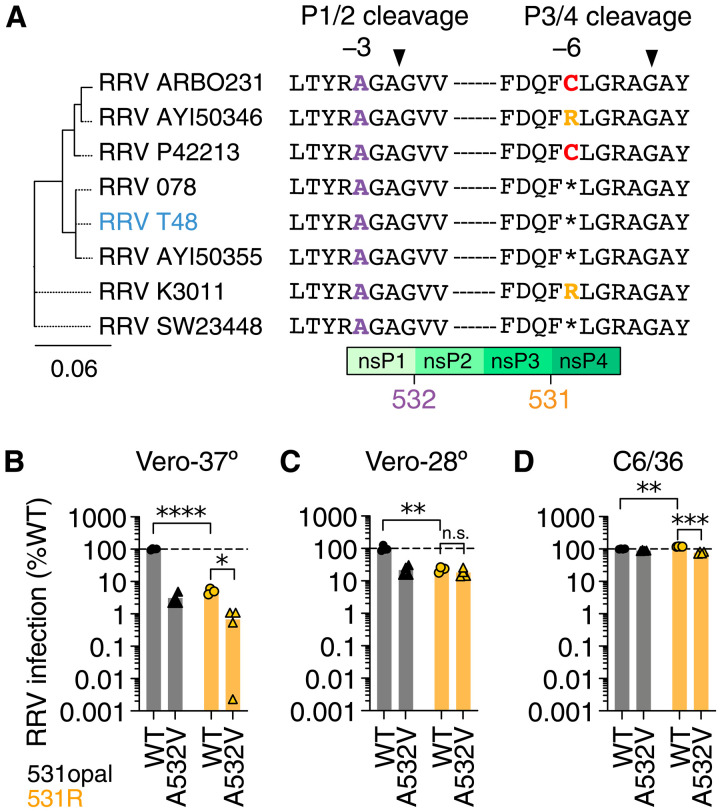
Conserved P1/2 cleavage site residue helps tolerate opal to sense codon mutation in RRV. (**A**) Multiple sequence alignment of P1/2 and P3/4 cleavage sites across eight representative RRV strains. The RRV laboratory strain T48 used in this study is highlighted in blue. Maximum likelihood tree was constructed using full-length RRV sequences. (**B** to **D**) WT (RRV T48/531opal) and variant (531R) RRV RNAs expressing mVenus was transfected into African green monkey (Vero) cells grown at either 37°C (B) or 28°C (C) and *Aedes* mosquito (C6/36) cells grown at 28°C (D). Cells were harvested 72 hours postinfection, and infection rates were quantified using flow cytometry and normalized to WT RRV (*n* = 4). One-way ANOVA with Tukey’s multiple comparisons test. *P < 0.05; **P < 0.01; ***P < 0.001; ****P < 0.0001; n.s., not significant.

To test this hypothesis, we focused on the RRV, a member of the SFV clade. We generated variants of RRV strain T48, carrying either opal (531opal) or arginine (531R) codons. We chose arginine due to its prevalence among RRV and other viruses within the SFV clade (Fig. 6A and fig. S10). We also introduced an nsP1 mutation (A532V), previously reported to reduce the P1/2 cleavage efficiency (*45*), into 531opal and 531R RRV backgrounds. We next quantified the growth of these four viruses in vertebrate (Vero-37° and Vero-28°) and mosquito (C6/36) cells.

Consistent with our SINV data, we found that the growth of RRV 531R was considerably lower than WT RRV T48 (531opal) in Vero-37° cells (Fig. 6, B to D) but was partially rescued in Vero-28° and fully rescued in C6/36 cells. If nsP1 A532V were a general hypomorphic mutation, we would expect it to be detrimental under all conditions tested. In contrast to this expectation, introducing the A532V mutation into WT RRV (531opal) also resulted in reduced growth in Vero-37°, which could be partially rescued in Vero-28° and C6/36 cells, respectively (Fig. 6, B to D). This temperature-sensitive replication phenotype of RRV A532V is consistent with previously described polyprotein processing mutants in SFV (*18*). In line with our hypothesis, introducing the A532V mutation in the RRV 531R background further reduced RRV 531R growth in Vero-37° (~7.5-fold) but had a less severe consequence in C6/36 (~1.5-fold) cells (Fig. 6, B and D). Our findings demonstrate that (similar to SINV and SFV), the nsP1 P1/2 cleavage site in RRV (especially the alanine residue at position 532) helps minimize the deleterious effects of sense codon mutations at the nsP3 opal codon site. Further reducing P1/2 cleavage efficiency via the A532V mutation impedes RRV 531R growth in Vero-37° but less so in Vero-28° or C6/36 cells. Evolutionary conservation of the P1/2 cleavage motif may thus explain the high incidence of opal-to-sense mutations across the SFV lineage. Collectively, our study establishes the mechanistic basis for the host (temperature)–specific selective constraint that leads to the retention of the opal codon in most dual-host alphaviruses.

## DISCUSSION

In this study, we investigate the biological cause underlying the widespread retention of the premature opal termination codon in nsP3 among alphaviruses. Using saturation mutagenesis and pooled selection, we comprehensively assessed all possible substitutions at this codon site in the prototype alphavirus SINV. These and subsequent experiments led us to conclude that host temperature is the predominant source of selective constraint at this site (Fig. 1), with the opal codon being significantly preferred over all others in Vero-37°. However, this preference is considerably lower at 28° in primate and mosquito cells. Unlike amber and ochre termination codons, the opal termination codon ensures the optimal degree of readthrough in Vero-37° that produces sufficient levels of the nsP4 viral polymerase (Fig. 2).

The opal codon also does not produce as much P1234 polyprotein as sense-codon variants, which disrupts SINV proteolytic processing cadence (Fig. 3 and 4). Lower passaging temperatures in vertebrate or mosquito cells increase translational readthrough, restoring alternate stop codons’ fitness. However, higher readthrough also leads to similar processing delays in WT, akin to sense-codon variants, lowering WT fitness so it is no longer superior to sense-codon variants (Fig. 3 and 4). Unexpectedly, we find that nsP processing defect in a sense-codon SINV variant in Vero-37° can be compensated by an additional delay in a subsequent step, which helps restore the proper orchestration of nsP processing (Fig. 5). Consistent with a previous report, such delays in polyprotein processing may benefit SINV replication in mosquito cells (Fig. 5H) (*46*).

We also extend our SINV findings to the distantly related RRV, a member of the SFV alphavirus clade, in which opal-to-sense mutations are present at relatively high frequencies (Fig. 6A and fig. S9). We show that, like SINV, an RRV sense-codon variant grows poorly in Vero-37°, but its growth is partially and fully recovered at 28° in primate and mosquito cells, respectively (Fig. 6, B to D). We also show that the P1/2 cleavage site in RRV and related old-world alphaviruses is naturally optimized to tolerate opal to sense codon mutations (figs. S8 and S9). Introducing any further delay in P1/2 cleavage significantly reduces the growth of the RRV sense-codon variant in vertebrate cells at 37°C but less so in vertebrate and mosquito cells at 28°C (Fig. 6, B and D). These results nicely recapitulate prior findings from the related SFV alphavirus, indicating that alphaviruses within the SFV clade use analogous strategies to tolerate the presence of sense codons at the opal codon site (*18*, *47*).

Our findings provide insights into how alphaviruses encoding opal to sense codon mutations exist and may even thrive in nature. Cysteine variants (the only naturally occurring opal-to-sense mutation found in SINV) exhibit fitness levels comparable to or exceeding those of WT SINV in mosquito cells. Another alphavirus, EEEV, also acquired opal-to-cysteine mutations upon passaging in mosquito cells (*14*). Our current results also show increased growth of cysteine (SINV) and arginine (RRV) mutants in mosquito cells (Figs. 1E and 6D). Therefore, we propose that opal-to-sense mutations spontaneously arise and are favored in the mosquito vector. It is premature to conclude that these mutations may be entirely enabled due to the lower host temperature because alphavirus replication in mosquito cells is far less understood and may differ from vertebrate cells in some key steps. Opal-to-sense mutations may play an adaptive role in this respect. Our results also demonstrate that the co-occurring P1/2 cleavage-site residues Thr^538^ (SINV-AR86) and Ala^532^ (RRV) are essential for the viability of opal to sense-codon mutants at high temperatures. Because both of these P1/2 polymorphisms have been demonstrated to blunt type I/II IFN responses in mammalian cells, they likely co-occur with opal-to-sense mutations to confer fitness advantages in both vertebrate and mosquito hosts (Figs. 5, C and H, and 6, B and D) (*42*, *43*, *45*, *47*).

Temperature is a critical environmental cue sensed by many microbial pathogens, including viruses, which navigate through multiple hosts, enabling them to instantaneously adapt their replicative strategies to their new host environment (*48*–*50*). Arboviruses like West Nile virus have been shown to encode molecular mechanisms that sense shifts in host temperature and adjust their replicative strategies (*49*). Temperature is especially critical for RNA viruses like alphaviruses because it can alter many fundamental aspects of their genome replication, transcription, and translation (*33*, *50*). Our findings suggest that the nsP3 opal codon adapts the replication of dual-host alphaviruses like SINV to host temperatures. Phylogenetic analysis of alphaviruses shows a correlation between the presence of the nsP3 opal codon and the alphaviruses’ ability to infect endothermic hosts or at high ambient temperatures (fig. S9A).

In contrast to the strong conservation of the opal codon in dual-host alphaviruses that navigate through insect and vertebrate hosts, alphaviruses that infect marine vertebrate fishes like salmon (salmonid alphavirus, Norwegian salmonid alphavirus, and salmon pancreatic disease virus), trout (sleeping disease virus), and hagfish (Wenling hagfish virus) encodes sense codons (arginine: CGA and CGG or alanine: GCU) instead. Given that these fishes live at near-freezing temperatures, it is likely that the high readthrough efficiency of the opal codon at such low temperatures renders it unnecessary. This is supported by the complete loss of the surrounding readthrough codon context, including the downstream cytosine base essential for optimal PRT (fig. S9B) (*8*, *51*, *52*). However, marine alphaviruses that infect endothermic mammals, e.g., seals (southern elephant seal virus) and porpoises (Alaskan harbor porpoise alphavirus) or tropical fish species like flounder and frogfish (Wenling fish alphavirus), still preserve the opal codon and the downstream cytosine, presumably because it still serves a valuable role at such high temperatures (fig. S9B). Because marine alphaviruses are considered an ancestrally branching clade, the nsP3 opal codon may thus represent an early innovation in alphavirus evolution (*53*).

Our hypothesis of temperature-dependent retention of the opal codon does not explain the near-universal preservation of the opal codon in insect-restricted alphaviruses, suggesting that additional insect host-specific selective constraints might exist in this alphaviral lineage. Most non-opal substitutions are depleted to a greater extent in mosquito cells than vertebrate cells at 28°C (Fig. 1, E and F), suggesting that, even for dual-host alphaviruses, additional host-specific differences influence mutational tolerance at this site. Although our in vitro model reveals mechanistic insights into the mutational constraints affecting the nsP3 opal codon, it may not fully account for additional constraints that could be present in vivo within vertebrate or mosquito host species (*54*, *55*). The marked fitness differences we observe between different sense-codon SINV variants may result from differences in the processing or functionality of nsP3 proteins encoding different amino acids at this position.

Our results support a model where the alphavirus nsP3 opal codon helps balance viral polymerase (RdRp) production and polyprotein processing efficiency, highlighting the importance of maintaining optimal concentrations of viral polyprotein precursors. Like alphaviruses, other RNA viruses with limited coding capacities, such as members of *Flaviviridae*, *Picornaviridae*, and *Coronavirinae* families, have evolved similar strategies of using polyprotein precursors to perform cellular functions that are distinct from the mature proteins (*56*–*58*). Among these viruses, proteolytic processing cadence is influenced by the abundance of polyprotein substrates, changes in substrate preference of the viral protease, or the interface and positioning of different cleavage sites (*59*–*62*). Our study showcases the effect of cell-intrinsic (host genetics) and extrinsic (temperature) factors on this critical process. In addition, our results highlight unexpected countermeasures viruses may evolve to restore processing efficiency.

Translational readthrough mechanisms also occur outside of vertebrate RNA viruses, particularly within some members of the alpha-like supergroup. These include plant viruses like tobraviruses, tobamoviruses, and furoviruses (*8*). These viruses also use PRT of “leaky” termination codons to synthesize their viral replicases (*28*, *63*). Our work suggests that the opal codon serves as a crude but effective thermometer for viral replication, indicating that deploying leaky termination codons might be a broad strategy to sense and respond to temperature stress, whether that is abiotic in the case of plant viruses and alphaviruses that infect ectothermic hosts, or biotic, in the case of host-switching alphaviruses infecting endothermic vertebrates.

## MATERIALS AND METHODS

### Insect and mammalian cell culture

African Green Monkey (*C. aethiops*)–derived Vero and BSC40 cells, HEK293T, and human hepatocyte epithelial (Huh7) cells were grown at 37°C under 5% CO_2_ in humidified incubators. C6/36 *A. albopictus* cells were grown at 28°C under 5% CO_2_ in humidified incubators and were cultured in high-glucose, l-glutamine minimal essential medium (Gibco) supplemented with 10% fetal bovine serum (Cytiva) and 1% penicillin-streptomycin (Gibco). All cells were cultured in high-glucose, l-glutamine minimal essential medium (Gibco) supplemented with 10% fetal bovine serum (Cytiva) and 1% penicillin-streptomycin (Gibco).

### Saturation mutagenesis screen

The opal variant library was generated via PCR mutagenesis. Briefly, a 3.8-kb template spanning P34 was PCR amplified from the WT TE3’2J:GFP infectious clone (primers in data S1). Mutagenic primers carrying ambiguous bases (NNN) at the opal codon site were used to generate the insert for downstream Gibson assembly. The TE3’2J:GFP viral backbone fragment lacking WT nsP3 was generated by digesting the infectious clone with *AgeI* and *BstEII* enzymes. Backbone and insert components were gel purified, mixed in 1:3 molar ratios, assembled via a two-part Gibson assembly (NEB HiFi assembly mix), and transformed into Endura DUO Electrocompetent Cells (LGC Bioresearch Technologies). Plasmid DNA was recovered from ~70,000 colonies collected and pooled from three independent replicates to ensure ~1000-fold coverage of the library of 64 variants. The entire screen was performed twice independently. Two micrograms of the *XhoI*-linearized variant library or WT TE3’2J:GFP plasmid was used for IVT reaction using SP6 RNA polymerase to generate pooled, capped viral RNA. In vitro, transcribed viral RNA was transfected into ~2 million cells using Lipofectamine LTX according to the manufacturer’s protocol (Thermo Fisher Scientific). Viral infection rates were assessed by monitoring GFP fluorescence, and supernatants were collected at 48 hpi in Vero cells grown at 37° and 28°C and 72 hpi in C6/36 cells grown at 28°C. The sequencing library was generated as follows: Virion RNA was isolated from collected supernatants using the QIAMP viral RNA isolation kit (Qiagen), reverse transcribed using Superscript III Reverse Transcriptase (Invitrogen), and an SINV-specific RT primer (5′-GTCGGATGATATTTCTCCAAAGGCGC-3′). Purified cDNA from postselection virion RNA or preselection library plasmid DNA was used to amplify the entire *AgeI/BstEII* flanked 3.8-kb region spanning the nsP3-nsP4 using KOD Hot-start Master Mix and the following cycling conditions: (1) 95°C 2 min, (2) 95°C 20 s, (3) 70°C 1 s (cool at 0.5°C/s), (4) 50°C 30 s, and (5) 70°C 40 s (repeat steps 2 to 5 24 times), 4°C hold. This PCR (PCR0) product was purified using AMPure beads and diluted to 0.5 ng/μl before generating unique molecular identifier (UMI)–tagged amplicons spanning the opal codon site. The UMI-tagging PCR1 using KOD Hot-start Master Mix was carried out with the following cycling conditions: (1) 95°C 2 min, (2) 95°C 20 s, (3) 70°C 1 s (cool at 0.5°C/s), (4) 50°C 20 s, (5) 70°C 20 s (repeat steps 2 to 5 nine times), and (6) 95°C 1 min, 4°C hold. UMI-tagged PCR1 was AMPure bead purified and diluted to bottleneck to 3 × 10^5^ barcodes, which corresponds to ≥3 reads per barcode at the target sequencing read depth. The final PCR2 step of the sequencing library preparation stage involved amplification using indexing primers with the following cycling conditions: (1) 95°C 2 min, (2) 95°C 20 s, (3) 70°C 1 s (cool at 0.5°C/s), (4) 55°C 20 s, and (5) 70°C 20 s (repeat steps 2 to 5 23x), 4°C hold. Last, all indexed PCR2 products were AMPure bead purified before and after gel purification and pooled before sample submission for next-generation sequencing. Sequencing was performed on an Illumina MiSeq Nano V3 at PE150, which generated ~21 million paired-end reads. Codon counts for each variant were determined from reads with sequence quality ≥ Q30 using the dmstools2 package (*64*).

### Virus constructs

All SINV and RRV stop codon mutant plasmids were generated via site-directed mutagenesis. Mutagenic primers were designed using Takara’s In-Fusion Cloning Primer Design tool (data S1). PCR reactions were performed using Phusion polymerase (NEB) with the following cycling conditions: (1) 98°C 30 s, (2) 98°C 30 s, (3) 50°C 30 s, (4) 72°C 7 min (repeat steps 2 to 4 18x), (5) 72°C 10 min, and (6) hold at 4°C. Reactions were then *DpnI*-treated for 6 hours and purified using the Monarch PCR and DNA Clean-up Kit (NEB) according to the manufacturer’s protocol. The purified reaction was then transformed into DH5α cells, and DNA was isolated using the Monarch Plasmid Miniprep Kit (NEB) according to the manufacturer’s protocol (see data S1 for specific protocols). Translation and translational readthrough reporters were generated by digesting parental and mutant plasmids and cloning in luciferase (Nano/Firefly) using Gibson assembly (NEB). The Gibson reactions were then transformed into DH5α cells, and DNA was isolated using the Monarch Plasmid Miniprep Kit (NEB) according to the manufacturer’s protocol.

### Independent viral growth assays

Two micrograms of infectious clones were linearized with *XhoI* (for SINV) and *SacI* (for RRV) (NEB) and subjected to IVT using SP6 RNA polymerase (NEB) according to the manufacturer’s suggested protocol. C6/36 and Vero cells were seeded into 24-well to 70 to 80% confluency and transfected with IVTs using Lipofectamine LTX (Thermo Fisher Scientific) according to the manufacturer’s protocol. Cells were collected 48 hours posttransfection and analyzed by flow cytometry (BD Fortessa). Infectious virus was quantified using end-point dilution median tissue culture infectious dose (TCID_50_) assays on Vero cells. All TCID_50_ assays were incubated at a permissive temperature (28°C), given the temperature-sensitive nature of SINV sense-codon variants.

### Viral competition assays

Genome copies of WT and mutant virus stocks were calculated using RT-qPCR. Briefly, cDNA synthesis was performed on viral supernatant using M-MuLV Reverse Transcriptase (NEB) with oligo(dT) (20mer+5′Phos) (IDT) according to the manufacturer’s protocol. RT-qPCR analysis was performed using SYBR Green master mix (Thermo Fisher Scientific) with gene-specific primers according to the manufacturer’s protocol and the Applied Bioscience StepOnePlus-qRT-PCR machine (Life Technologies). Once obtained, WT and mutant SINV virus stocks were normalized to equal genome copies. C6/36 and Vero cells were seeded into 24 wells and infected with TE3’2J-mCherry WT virus alongside TE3’2J:GFP WT or mutant viruses at a 1:1 ratio [multiplicity of infection (MOI) = 0.1]. Cells were collected 48 hpi and analyzed by flow cytometry (BD Fortessa) to determine the percentage of cells infected with WT (red) and variant (green) viruses.

### Total RNA extractions and real-time RT-qPCR analysis

To quantify minus-strand RNA copies, cells were seeded into a 24-well and infected with WT or variants at >90% confluency at MOI = 5. At 4 hpi, cells were harvested using TRIzol reagent (Thermo Fisher Scientific), and RNA was extracted using the Direct-zol RNA Miniprep kit (Zymo) according to the manufacturer’s protocol. Following RNA extraction, cDNA was synthesized using M-MuLV Reverse Transcriptase (NEB) with minus-strand specific primers (IDT). To quantify plus-strand RNA copies, cells were seeded into a 24-well and infected with WT or variant SINV at >90% confluency at MOI = 0.1. Cells were then harvested 18 hpi using TRIzol reagent (Thermo Fisher Scientific). RNA was extracted using the Direct-zol RNA Miniprep kit (Zymo) according to the manufacturer’s protocol. Following RNA extraction, cDNA was synthesized using M-MuLV Reverse Transcriptase (NEB) with gene-specific primers and oligo(dT) (20mer+5′Phos) (IDT) according to the manufacturer’s protocol. In all cases, RT-qPCR analysis was performed using SYBR Green master mix (Thermo Fisher Scientific) with plus-strand specific primers according to the manufacturer’s protocol and using the Applied Bioscience StepOnePlus-qRT-PCR machine (Life Technologies).

### Viral translation assays

SINV nsP3-Nluc reporter viruses (data S1) were generated by digesting 2 μg of plasmid DNA with *XhoI* and performing IVT using SP6 RNA polymerase (NEB) according to the manufacturer’s protocol. C6/36 and Vero cells were seeded into black-walled, clear-bottom 96-well plates at 70 to 80% confluency and transfected using Lipofectamine LTX (Thermo Fisher Scientific) according to the manufacturer’s protocol. At indicated times posttransfection or infection, translation was quantified using the NanoGlo luciferase assay system (Promega) according to the manufacturer’s protocol. Luminescence was recorded using a Cytation3 Imaging Reader (BioTek).

### Translational readthrough assay

C6/36 and Vero cells were seeded into black-walled, clear-bottom 96 wells at 70 to 80% confluency and transfected with the dual-reporter translational readthrough constructs (data S1) using Lipofectamine LTX (Thermo Fisher Scientific) according to the manufacturer’s protocol. At 48 hours posttransfection, Fluc and Nluc signals were quantified using the Nano-Glo Dual luciferase assay system (Promega) according to the manufacturer’s protocol. Luminescence was recorded using a Cytation3 Imaging Reader (BioTek). Fluc-to-Nluc ratios obtained for each stop codon variant were compared to a control construct carrying a sense mutation in place of the opal, representing a 100% translational readthrough.

### IP and Western blot analysis

C6/36 and Vero cells were seeded into six-well plates and infected at >90% confluency (MOI = 5). Cells were washed with cold 1X PBS and harvested using radioimmunoprecipitation assay (RIPA) buffer (Pierce) supplemented with 1X protease inhibitor (cOmplete). Protein was denatured using 4X Laemmli buffer (Bio-Rad) with 10% β-mercaptoethanol (Sigma-Aldrich). Western blots were run on Mini-PROTEAN precast gels and then transferred to a Trans-Blot 0.2-μm nitrocellulose membrane using the Trans-Blot Turbo Transfer System (Bio-Rad). Blots were incubated in 5% bovine serum albumin and either primary anti-FLAG polyclonal antibody (Proteintech, Thomas Scientific, 20543-1-AP) at 1:3000, anti-nsP2 antisera at 1:3000, antinsP4 antisera at 1:2000, anti-H3 antibody (Abcam, ab1791) at 1:3000, or anti-β actin antibody (Abcam, ab8224) at 1:3000 dilution overnight at 4°C and washed with 1X TBS with 0.1% Tween 20. The blots were then probed with secondary anti-rabbit HRP (horseradish peroxidase) conjugate (R&D Systems). Blots were visualized using a Bio-Rad ChemiDoc Imaging System. Western blots probing for nsP (nsP2, nsP3, and nsP4) expression and polyprotein processing were performed with independent biological replicates for each virus under different host conditions. Densitometric quantification of nsP3 and P34 products was performed using replicate data (fig. S6) with ImageJ.

C6/36 and Vero cells were seeded at >90% confluency. Approximately 3 million cells were infected with WT or sense-codon variants at MOI ≥ 5. Viral adsorption was carried out for 30 min at 4°C. The inoculum was removed, and infection was synchronized with the addition of prewarmed media. At indicated times postinfection, cells were washed twice with PBS and collected following trypsinization (Vero) or manual dislodgement (C6/36). Collected cell pellets were frozen at −80°C until further use. For IP, frozen cell pellets were thawed and resuspended in 100 μl of RIPA lysis buffer (Pierce) supplemented with a protease inhibitor cocktail (cOmplete). Cell lysis was carried out on a rotating platform for 1 hour at 4°C, following a brief vortexing step. Collected cell lysates were spun down at 14,000*g* for 15 min at 4°C to remove cell debris. Protein concentrations in the clarified lysates were determined using a Pierce BCA protein assay kit (Thermo Fisher Scientific). For each sample, 50 μg of protein was used as the input, whereas 50 μg of protein was used for subsequent IP using Pierce anti-FLAG magnetic agarose beads (Thermo Fisher Scientific) according to the manufacturer’s protocol. Briefly, 50 μl of beads prewashed with RIPA buffer was used to immunoprecipitate 50 μg of lysate. IP was carried out for 2 hours on a rotating platform at 4°C. Beads were washed twice on a magnetic rack with lysis buffer. Bound protein was eluted from the beads using purified 3X FLAG peptide (Sigma-Aldrich) by shaking (1400 rpm) at room temperature for 30 min according to the manufacturer’s protocol. For final elution, samples were placed on a magnetic rack and eluted in 50 μl of lysis buffer. Protein was denatured using 4X Laemmli buffer (Bio-Rad) with 10% β-mercaptoethanol (Sigma-Aldrich). Western blot analysis and densitometric quantification of total and immunoprecipitated protein were carried out as described above.

### Phylogenetics and sequence analysis

A multiple sequence alignment of nsP4 coding sequences from 49 extant alphavirus species was constructed using Clustal Omega. Alignments were manually curated using Geneious and maximum likelihood phylogenetic trees, generated using the HKY85 substitution model in PHYML, using 100 bootstrap replicates for statistical support. Phylogenetic trees were visualized using FigTree. Logo plots were generated using Skylign (*65*). Transcriptome-weighted codon usage counts for African green monkey (*C. aethiops*) and Asian tiger mosquito (*A. albopictus*) derived from available GenBank and RefSeq data were obtained from CoCoPUT (https://dnahive.fda.gov/) (*23*, *24*).

### Statistics

Statistical analyses were conducted using GraphPad Prism (v10.2, GraphPad Software Inc., San Diego, CA). Data were first checked for a normal (Gaussian) distribution using the Kolmogorov-Smirnov test. For data meeting normality (α = 0.05) and similarity of variance criteria, means were compared using, unless otherwise stated, two-way analysis of variance (ANOVA) (for multiple groups with two variables) with post hoc tests used for multiple comparisons.
