# Supplementary material for: A conserved opal termination codon optimizes a temperature-dependent trade-off between protein production and processing in alphaviruses
Source: Sci Adv. 2025 Apr 18;11(16):eads7933. doi: 10.1126/sciadv.ads7933 (PMC13105319; doi:10.1126/sciadv.ads7933)
Supplement: Supplementary file 1 — Figs. S1 to S10 Legends for data S1 to S3 [file sciadv.ads7933_sm.pdf]

Science Advances
AAAS

Supplementary Materials for

A conserved opal termination codon optimizes a temperature-dependent trade-off between protein production and processing in alphaviruses

Tamanash Bhattacharya *et al.*

Corresponding author: Tamanash Bhattacharya, tbhattac@fredhutch.org

Sci. Adv. **11**, eads7933 (2025)
DOI: 10.1126/sciadv.ads7933

The PDF file includes:

Figs. S1 to S10
Legends for data S1 to S3

Other Supplementary Material for this manuscript includes the following:

Data S1 to S3

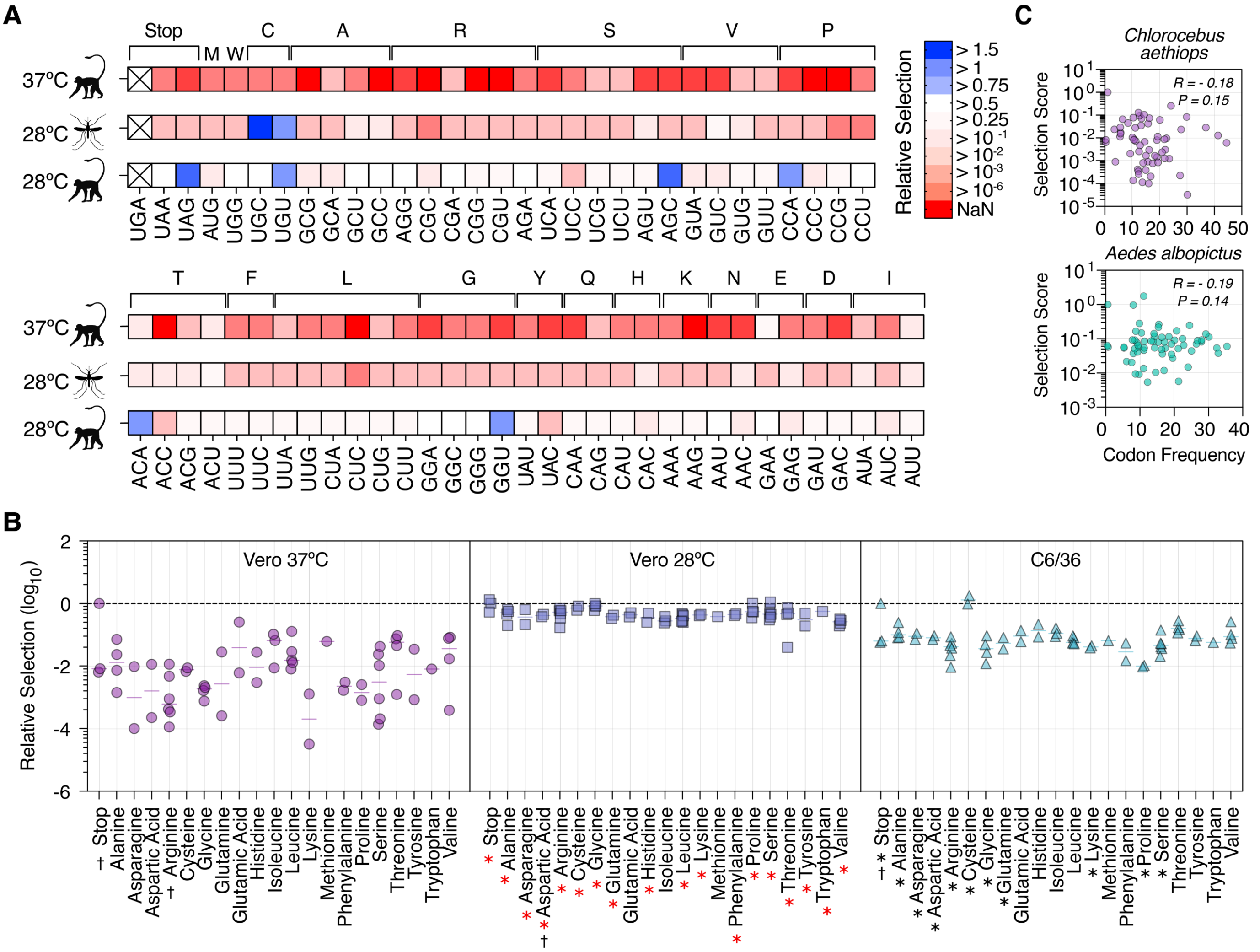

Fig. S1. ***Selection scores of codon variants across cell types and temperatures.*** (A) Heat-map of selection scores of all codon variants, grouped based on encoded amino acids (on top). (B) Mean selection scores of SINV variants encoding different amino acids. The dotted line represents the selection score of WT (opal) SINV. Each data point is a synonymous codon encoding the corresponding amino acid. † denotes statistically significant different selection scores between synonymous codons in the same condition. Red asterisks indicate statistically significant differences between Vero-37º and Vero-28º cells, whereas black asterisks indicate statistically significant differences between Vero-37º and C6/36 cells. Two-way ANOVA with Tukey's test for multiple comparisons. Pairwise unpaired t-tests with Welch's correction. (C) Pearson's correlation between selection scores and codon usage frequencies in African green monkey (source of Vero cells) and *Aedes albopictus* (source of C6/36 cells).

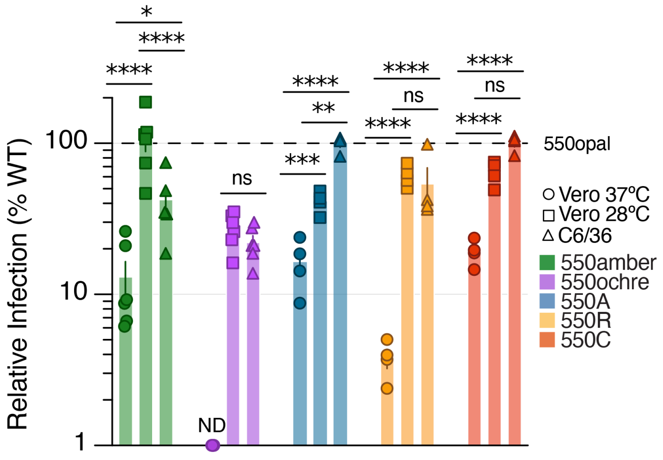

Fig. S2. ***Fitness of alternate stop and sense-codon SINV variants.*** Alternate-stop codon variants (550amber and 550ochre) and sense-codon variants (550A, 550R, 550C) were engineered into a SINV-GFP reporter virus. Horizontal dotted line represents infection rates of wildtype SINV (550opal). Independent growth of variants was tested by infecting Vero- 37º, Vero- 28º, or C6/36 cells at MOI of 0.1 and quantifying GFP-expressing cells via flow cytometry 48 hours post-infection (n=4-6). Relative infection rates are normalized to WT SINV (550opal). Two-way ANOVA with Tukey's multiple comparisons test. **** = P < 0.0001, *** = P < 0.001, ** = P < 0.01, * = P < 0.05, ns = not significant, ND – not detected.

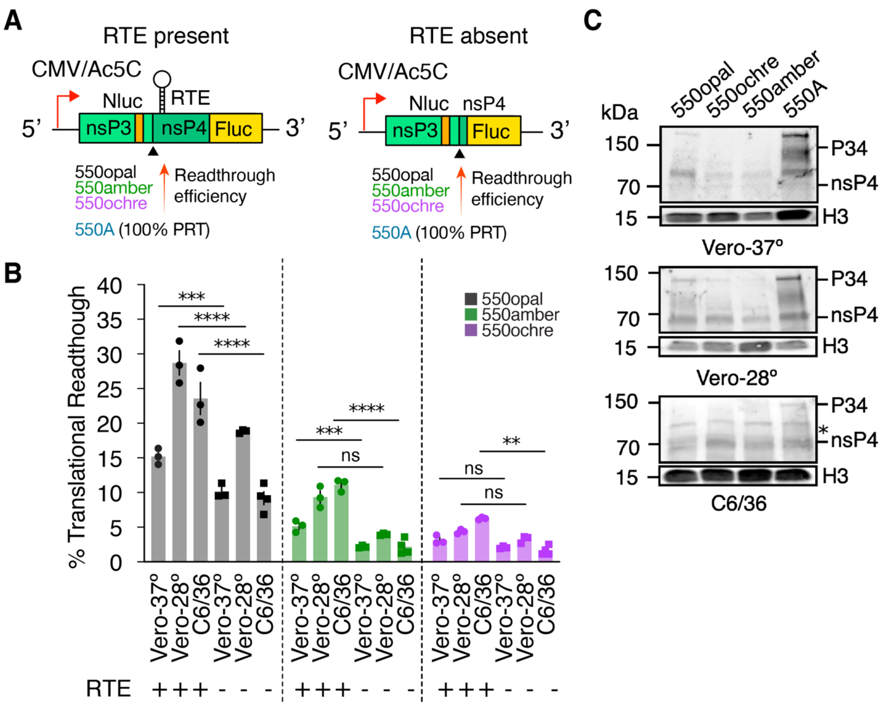

Fig. S3. A termination codon readthrough element (RTE) is required for the translational readthrough in vertebrate and mosquito cells. (A) Translational readthrough constructs were designed to include (top) or exclude (bottom) the downstream RNA structure, known as the termination codon readthrough element (RTE). (B) Using reporters, translational readthrough was quantified relative to a reporter encoding a sense codon (GCA), which does not require translational readthrough for nsP4 expression. Two-way ANOVA with Sidak's multiple comparisons test. **** = P < 0.0001, *** = P < 0.001, ** = P < 0.01, ns = not significant. (C) Vero-37º, Vero-28º, or C6/36 cells were infected with WT (550opal), 550amber, 550ochre, or 550A sense-codon SINV variants (MOI=5). 18h post-infection, total protein was extracted for western blotting. Blots were probed with polyclonal sera against anti-nsP4, and anti-H3 monoclonal antibody. A non-specific band observed in C6/36 cell lysates is highlighted with an asterisk (*).

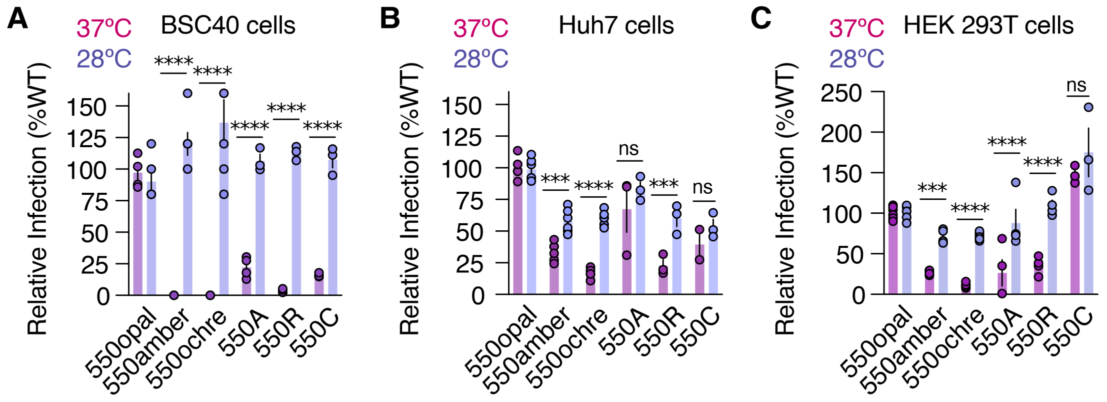

Fig. S4. Infection rates of alternate stop-codon and sense-codon SINV variants in different primate cells grown at either 37ºC or 28ºC. We transfected stop (550opal, 550amber, 550ochre), or sense-codon (550A, 550R, or 550C) SINV variants expressing GFP into African green monkey (BSC40) cells (A), Human hepatocyte epithelial (Huh7) (B), or Human embryonic kidney (HEK 293T) cells (C) grown at either 37ºC or 28ºC. Cells were harvested 48 hours post-infection, and infection rates were quantified using flow cytometry (n=3-6). Two-way ANOVA with Tukey's multiple comparisons test. **** = P < 0.0001, ** = P < 0.01, * = P < 0.05, ns = not significant.

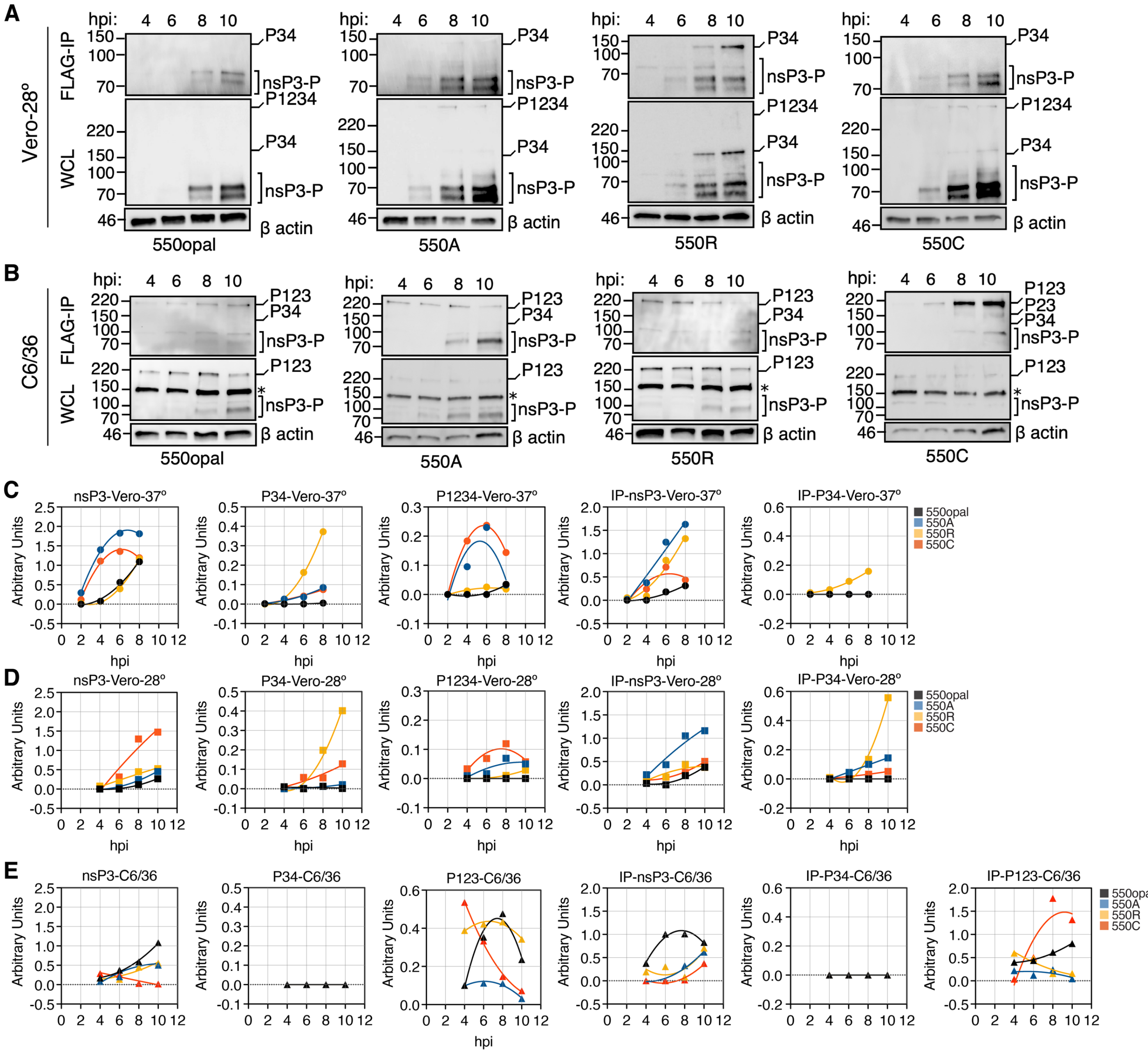

Fig. S5. Sense-codon variants exhibit polyprotein processing defects early during infection. WT (550opal) or sense-codon (550A, 550R, or 550C) SINV expressing 3XFLAG- tagged nsP3 were used to infect Vero-28º (B) or C6/36 (C) cells. At indicated times post-infection, total protein (WCL, whole cell lysate) was subjected to immunoprecipitation using anti-FLAG (FLAG-IP) to enrich for unprocessed and processed nsP3. Blots were probed with anti-FLAG and anti-β actin monoclonal antibodies. Lane denoted with an asterisk (*) represents a non-specific band observed in C6/36 cell lysates probed with anti-FLAG antibody. Note that this band is absent from the FLAG-IP fraction. (C-E) Using ImageJ software, we quantified processed nsP3 and unprocessed P34 via densitometry. Band intensities were normalized to the β actin loading control. Temporal trends in protein levels are visualized using second-order polynomial regression curves.

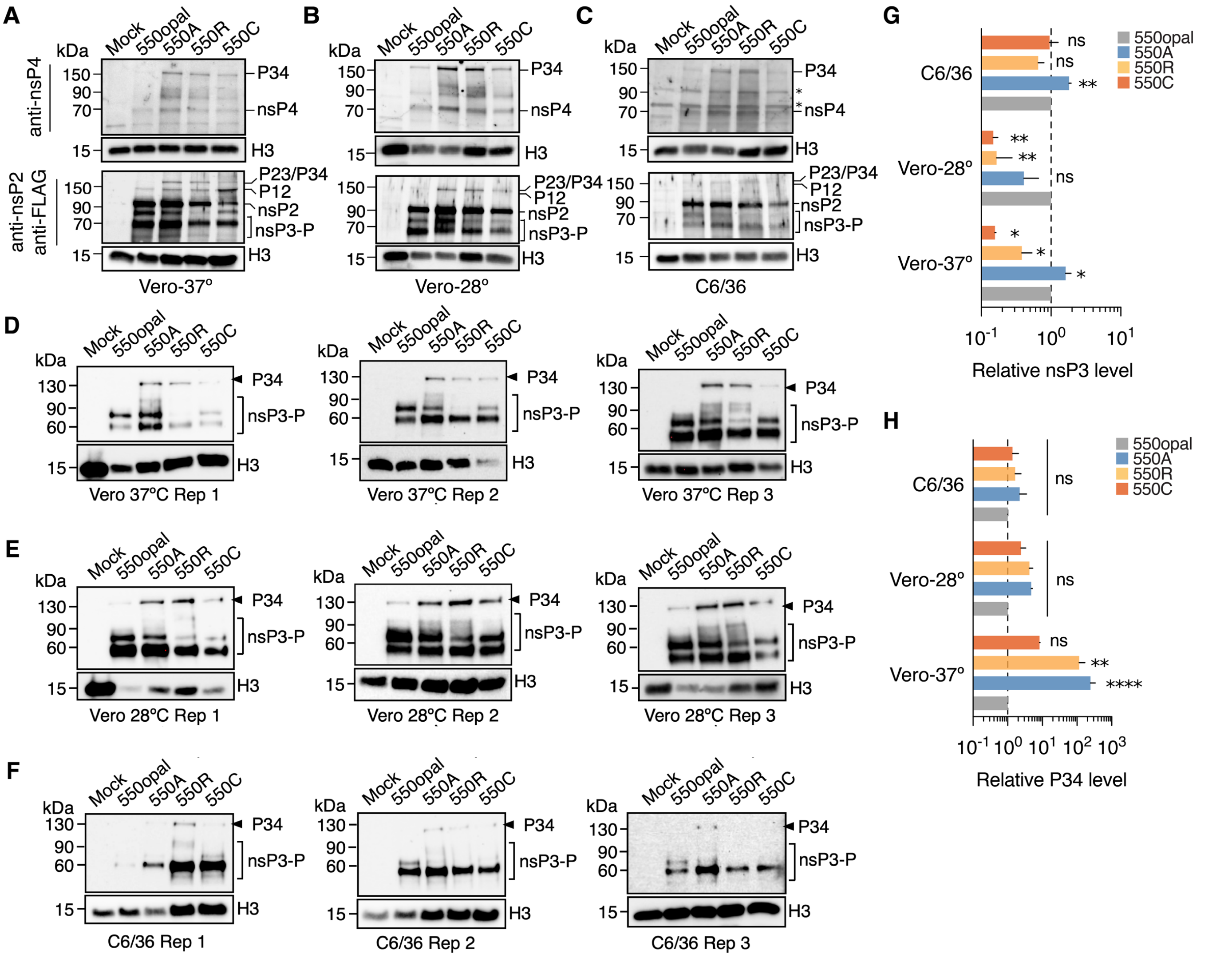

Fig. S6. The presence of sense codons at the nsP3 opal codon site impairs alphavirus nonstructural polyprotein processing. (A-C) WT (550opal) or sense-codon (550A, 550R, or 550C) SINV expressing 3XFLAG- tagged nsP3 were used to infect Vero-37º (A), Vero-28º (B) or C6/36 (C) cells. 18h post-infection, proteins were extracted and analyzed via western blotting using polyclonal sera against nsP4 (top) or a cocktail of nsP2 polyclonal serum and anti-FLAG polyclonal antibody (bottom). anti-histone H3 monoclonal antibody was used as loading control. Data are representative of three independent experiments. Lane denoted with an asterisk (*) represents non-specific bands observed in C6/36 cell lysates probed with anti-nsP4 polyclonal serum. (D-F) Replicate data of western blots used for quantification. 18h post-infection, proteins were extracted and analyzed via western blotting using anti-FLAG polyclonal antibody. Loading control was detected by anti-histone H3 monoclonal antibody. (G-H) Quantification of data shown in panels D-F. Levels of processed nsP3 (G) and unprocessed P34 (H) were quantified using ImageJ. Band intensities were normalized to the loading control H3. Two-way ANOVA with Dunnett's multiple comparisons test. **** = $P < 0.0001$, ** = $P < 0.01$, * = $P < 0.05$, ns = not significant.

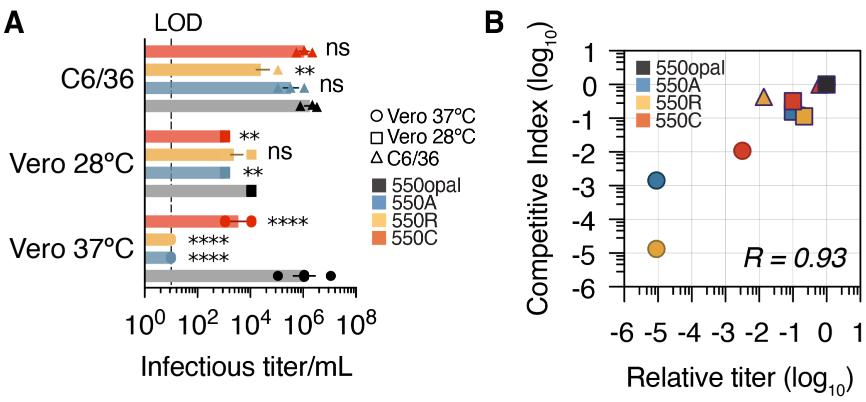

Fig. S7. Infectious virus production in sense-codon SINV variants. (A) WT (550opal), or sense-codon (550A, 550R, or 550C) SINV variants were transfected into African green monkey (Vero) cells grown at either 37ºC or 28ºC and Aedes mosquito (C6/36) cells at 28ºC. Viral supernatants were harvested 48 hours post-infection, and infectious virus was quantified using $TCID_{50}$ assays on Vero cells incubated at 28ºC (n=3). Dotted line represents the limit of detection (LOD) of the $TCID_{50}$ assay. Two-way ANOVA with Tukey's multiple comparisons test. **** = $P < 0.0001$, ** = $P < 0.01$, * = $P < 0.05$, ns = not significant. (B) Pearson's correlation test was performed comparing viral competitive index (Figure 1F) and relative infectious titer. Two-tailed P-value <0.0001.

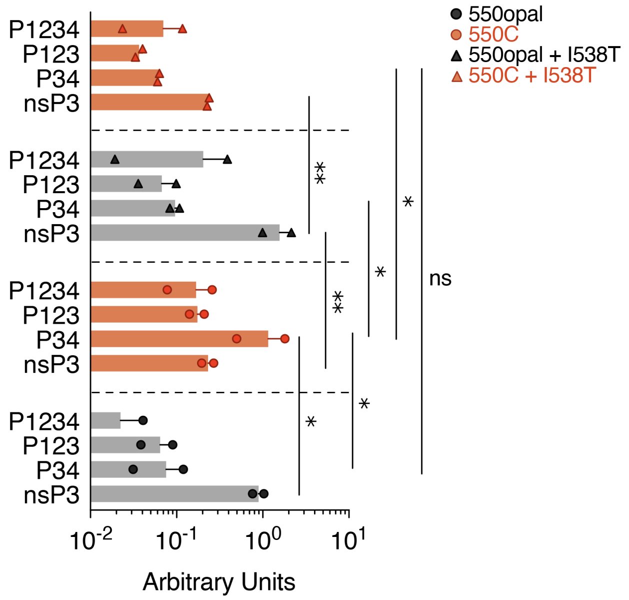

Fig. S8. Quantification of processed and unprocessed polyprotein in cells infected with wildtype and sense-codon SINV variant with and without I538T. WT (550opal), sense-codon (550C) and I538T-carrying variants (opal+I538T and 550C+I538T) expressing 3XFLAG-tagged nsP3 were used to infect Vero-37° cells. 18h post-infection, proteins were extracted and analyzed via western blotting using anti-FLAG polyclonal, and anti-histone H3 monoclonal antibodies. Using ImageJ software, we quantified processed nsP3 or unprocessed P1234, P123 and P34 using densitometry. Band intensities were normalized to the loading control H3. Quantified data represent the mean of two independent experiments. Two-way ANOVA with Tukey's multi comparisons test. ** = $P < 0.01$, * = $P < 0.05$.

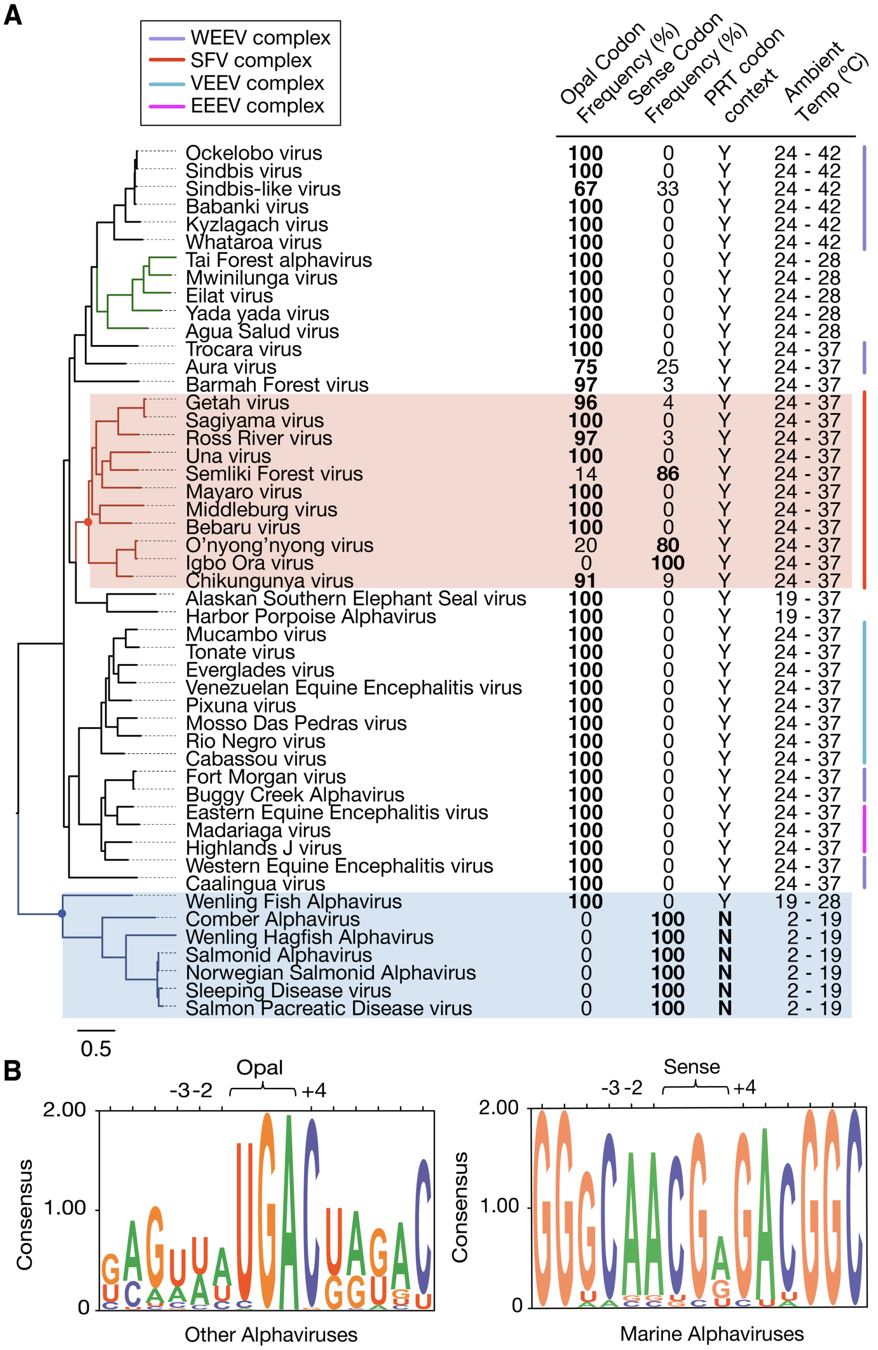

Fig S9. Phylogenetic conservation of the nsp3 opal codon in alphaviruses correlates with ambient host temperature. (A) Maximum likelihood tree of 49 extant alphaviruses. Viruses indicated in bold are described in this study. Semliki forest virus clade is highlighted in light red, insect-restricted alphavirus clade is highlighted in green and marine, vertebrate-restricted alphavirus clade is highlighted in blue. Nodes leading to lineages that exhibit a high frequency of opal to sense mutations are highlighted with solid circles. Columns next to each tip indicate the following: (First column) the percentage of sequenced isolates containing an opal stop codon, (Second column) the percentage of sequenced isolates containing a sense codon, (Third column) preservation of the surrounding codon context necessary for opal codon readthrough, (Fourth column) temperature ranges of the vertebrate or invertebrate hosts associated with each alphavirus species. (B) Logo plots show conservation at the opal stop codon site and its genomic context among terrestrial (top) and marine (bottom) alphaviruses. -3 and +4 positions indicate constrained residues that aid in the translational readthrough of an opal codon.

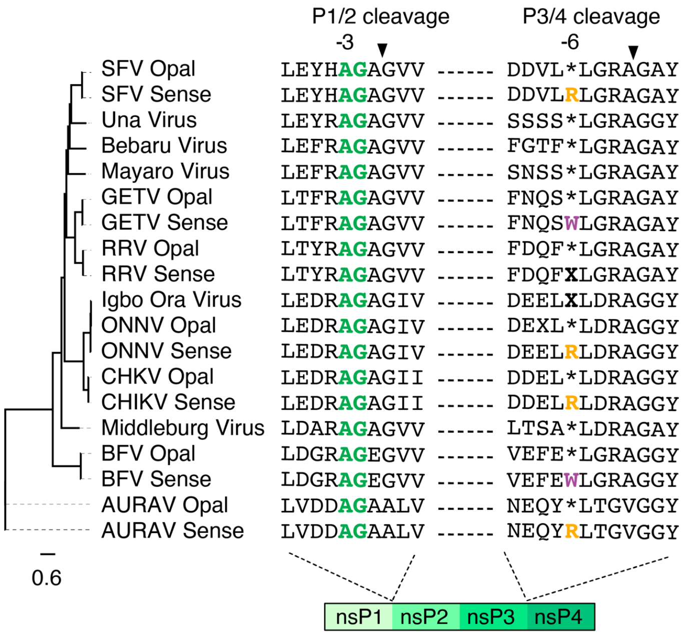

Fig S10. Conservation of P1/2 cleavage site residues among viruses within the SFV clade. Multiple sequence alignment of nsP1/2 and nsP3/4 cleavage site regions was generated using ClustalOmega. Maximum likelihood tree was generated from full-length consensus sequences derived from multiple sequence alignments.

Data S1. Construct and Primer Table used in this study

Data S2. Selection scores for variants collected under experimental conditions described in the study and correlation between selection scores and codon usage frequency

Data S3. Raw data for small sample sizes
